# Pancreatic exocrine damage induces beta cell stress in zebrafish larvae

**DOI:** 10.1007/s00125-025-06432-4

**Published:** 2025-04-28

**Authors:** Noura Faraj, Willem M. H. Hoogaars, B. H. Peter Duinkerken, Anouk H. G. Wolters, Kim Kats, Mette C. Dekkers, Arnaud Zaldumbide, Ben N. G. Giepmans

**Affiliations:** 1 Department of Biomedical Sciences, University Medical Center Groningen, University of Groningen, Groningen, the Netherlands; 2 Department of Cell and Chemical Biology, Leiden University Medical Center, Leiden, the Netherlands

**Keywords:** Beta cell function, Beta cell stress, Diabetes, Exocrine damage, Zebrafish larvae

## Abstract

**Aims/hypothesis:**

Excessive endoplasmic reticulum (ER) stress in beta cells can impair proliferation and contribute to autoimmune responses such as the destruction of beta cells in type 1 diabetes. Exocrine–beta cell interactions affect beta cell growth and function. Notably, exocrine abnormalities are frequently observed alongside overloaded beta cells in different types of diabetes, suggesting that exocrine stress may induce beta cell ER stress and loss. While a cause–consequence relationship between exocrine stress and beta cell function cannot be addressed in humans, it can be studied in a zebrafish model. Larvae develop a pancreas with a human-like morphology by 120 h post-fertilisation, providing a valuable dynamic model for studying pancreatic interactions. Our aim was to target exocrine cells specifically and address beta cell status using transgenic zebrafish models and reporters.

**Methods:**

To explore the impact of exocrine damage on beta cell fitness, we generated a novel zebrafish model allowing exocrine pancreas ablation, using a nifurpirinol–nitroreductase system. We subsequently assessed the in vivo effects on beta cells by live-monitoring dynamic cellular events, such as ER stress, apoptosis and changes in beta cell number and volume.

**Results:**

Exocrine damage in zebrafish decreased pancreas volume by approximately 50% and changed its morphology. The resulting exocrine damage induced ER stress in 60–90% of beta cells and resulted in a ~50% reduction in their number.

**Conclusions/interpretation:**

The zebrafish model provides a robust platform for investigating the interplay between exocrine cells and beta cells, thereby enhancing further insights into the mechanisms driving pancreatic diseases such as type 1 diabetes.

**Graphical Abstract:**

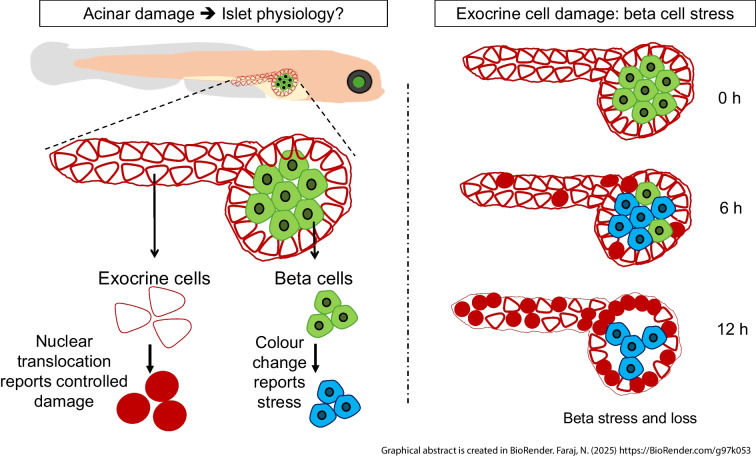

**Supplementary Information:**

The online version of this article (10.1007/s00125-025-06432-4) contains peer-reviewed but unedited supplementary material.


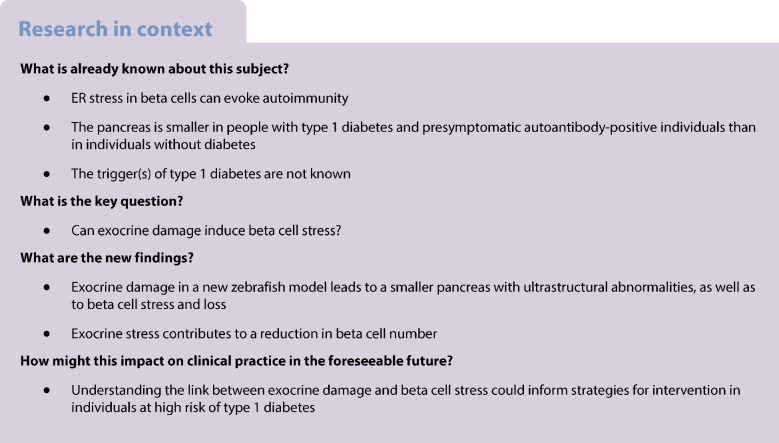


## Introduction

The pancreas is one organ with two major distinct functions, namely physiological regulated exocrine and endocrine secretion. The integrated infrastructure underscores the coherence between the islets of Langerhans and exocrine cells, which is vital for both normal function and disease [1, 2]. Beta cells within the islet regulate glucose homeostasis through insulin release in response to high blood glucose levels. However, the production of around one million insulin molecules per minute makes beta cells susceptible to endoplasmic reticulum (ER) stress [3]. Prolonged ER stress may lead to the accumulation of misfolded or aberrantly processed proteins, which can provoke an immune response and contribute to the development of type 1 diabetes, of which the trigger is not known [4].

Exocrine damage has been suggested as a potential trigger for beta cell dysfunction in different types of diabetes [5, 6]. For instance, individuals with type 1 diabetes and presymptomatic autoantibody-positive individuals have smaller pancreases than individuals without diabetes [7] and show signs of inflammation and stress in intermediate endocrine and exocrine cells [8, 9]. This suggests that generalised pancreatic malfunction occurs during and possibly pre the onset of type 1 diabetes and may contribute to the loss of beta cell function. Furthermore, exocrine dysfunction and subsequent beta cell loss is also observed in other types of diabetes, such as MODY, diabetes associated with pancreatitis and cystic fibrosis-related diabetes [10–12]. However, it remains unclear whether exocrine damage directly contributes to beta cell stress and loss.

Studies in humans do not allow for direct assessment of cause-and-effect or for real-time tracking of cellular interactions between exocrine and beta cells. The pancreas in zebrafish larvae is ideal for dynamic studies due to the larvae’s small size, transparency and compatibility for optical imaging [13]. The zebrafish pancreas develops from two buds, eventually acquiring a similar structure and function to the human pancreas. The principal islets of Langerhans forms by 24 h post-fertilisation (hpf) and becomes functional by 48 hpf. Exocrine and ductal cells develop through two stages, beginning at 72 hpf, encircling the islet and expressing pro-enzymes. By 120 hpf, the primary islet is well vascularised to sense blood glucose levels and innervated to enhance cellular activity [14–16]. Exocrine cells start to function by secreting proteases by 120 hpf and phospholipases by 144 hpf [17]. This study aimed to develop a novel zebrafish model to induce exocrine damage and track its effects on live beta cells using proper readouts such as ER stress and apoptosis.

## Methods

### Cloning and transgenic lines

The sequences for plasmids used to generate transgenic zebrafish lines are provided in the electronic supplementary material (ESM) Methods. All transgenic zebrafish lines were generated using the Tol2 transposon system [18] (ESM Tables 1, 2) on AB background (Central Animal Facility, University Medical Center Groningen, the Netherlands, ZDB-GENO-960809-7, https://zfin.org/ZDB-GENO-960809-7). The pT2-L200R150-MCS1 (pT2) construct was kindly gifted by K. Kawakami, Japan [19, 20], and the pT2*ef1α xbp1-gfp* plasmid was a kind gift from S. J. Du [21]. Details of all used Addgene plasmids and primers are given in ESM Tables 3, 4. The *t41q/n71s/f124t* triple mutant *ntr* (*ntr* is also known as *nfsB*) was generated according to [22]. The 3.5 *ubb:ANXA5-mvenus* was kindly gifted by T. van Ham [23]. The 995 bp upstream fragment of the *insulin* gene was extracted according to the protocol described in [24] using proper primers [25]. For the creation of transgenic lines or for transient expression studies, 25 pg DNA construct was micro-injected into single-cell embryos (Wild type AB zebrafish, Central Animal Facility, University Medical Center Groningen, the Netherlands, ZDB-GENO-960809-7, https://zfin.org/ZDB-GENO-960809-7).

The Tg(*insulin:gfp*) line was a kind gift from B. Peers: Tg(*insulin:gfp*) ulg021 Tg, Laboratory of Zebrafish Development and Disease Models (ZDDM), Belgium, ZDB-GENO-180604-1, https://zfin.org/action/genotype/view/ZDB-GENO-180604-1 [26].

### Zebrafish husbandry and maintenance

Wild-type AB and transgenic zebrafish were kept and raised in the Central Animal Facility (CDP) of the University Medical Center Groningen (UMCG) at 28°C. All procedures (feeding, environment, maintenance, etc.) were conducted according to the European and Dutch animal welfare legislation. Embryos were collected in the morning after separator removal in diluted medium of × 60 E3 (g/l: 17 NaCl, 0.80 KCl, 2.9 CaCl_2_ 2H_2_O, and 4.9 MgCl_2_ 6H_2_O). After 24 hpf, 200 µmol/l of 1-phenyl 2-thiourea (PTU) (Sigma-Aldrich, 103-85-5) was added to prevent pigmentation, and non-viable embryos were removed. To perform experiments, larvae were sedated with 200 mg/l ethyl 3-aminobenzoatemethanesulphonate (MS-222/Tricaine; Sigma-Aldrich, 886-86-2) and euthanised on ice with 4 g/l Tricaine no later than 120 hpf. Samples were randomly selected and assigned for treatment and experiments in a blinded manner. Only non-viable embryos and malformed embryos or larvae were excluded.

### In vivo NFP–NTR ablation

Single or double Tg(*ela3l:ntr*) zebrafish larvae were sedated with Tricaine and treated with 5 µmol/l NFP (LGC Standards, 20022446) or 0.1% DMSO (Sigma-Aldrich, 15,493-8) as a control unless stated otherwise. For imaging, larvae were embedded in 1% low-melting agarose (Alfa Aesar, 9012-36-6). In all experiments, NFP was not washed away, even during imaging.

### NFP toxicity

The viability of DMSO- or NFP-treated Tg(*ela3l:ntr*) zebrafish larvae was followed under a stereoscope at intervals of 3 h over a 12 h period following treatment with a gradient of increasing NFP concentrations. Zebrafish viability was determined based on the presence/absence of heartbeats and their quick swimming response to a touch stimulus applied to the tail (response to stimuli).

### Confocal imaging

Zebrafish larvae were embedded in 1% low-melting agarose using 35 mm glass bottom dishes (Greiner bio-one). Confocal imaging was conducted with a Zeiss LSM 780 NLO CLSM (Jena, Germany) or Leica SP8X (Mannheim, Germany) and frame acquisition with the proper excitation wavelengths. Maximum intensity projections are presented. Images were analysed using Fiji (2.14.0) [27, 28].

In vivo imaging was conducted at 1 h intervals for 12 h using a Zeiss CD7/CLSM900 microscope (Jena, Germany) to enhance resolution with the proper excitation wavelengths.

### Light sheet confocal imaging

Zebrafish larvae were sedated with Tricaine and immobilised in 1–1.5% low-melting agarose within capillaries. Images were acquired at hourly intervals using a Zeiss LS7 light sheet microscope (Jena, Germany) with × 5/0.1 illumination optics and the laser set at the proper excitation wavelength and z stack images were acquired at 1 or 2 µm intervals.

### Analysis of zebrafish pancreas

3D volumes were generated via Imaris (9.7.2) (Oxford Instruments) with default settings for isosurface rendering (ESM Video 1; available via http://www.nanotomy.org/OA/Faraj2025Diabetologia/). The pancreas length of zebrafish larvae was measured and normalised as a ratio to the whole larvae length. The images were captured using a Leica SP8X and mosaic images were merged using LAS X software. Length measurement was performed with Fiji.

### Histological analysis

Larvae at 120 hpf were processed following established protocols [29, 30] with some modifications. Briefly, zebrafish larvae were fixed overnight at 4°C in 10% neutral buffered formalin (Formaldehyde solution about 37%, Sigma-Aldrich, 1.4003.100). After fixation, zebrafish larvae were embedded and oriented on their lateral sides in 1% agarose blocks under a stereoscope. Samples were placed in ethanol 70% overnight at room temperature followed by dehydration in a series of ethanol gradients (70%, 96%, 100%). Ethanol was cleared in xylene and infiltration with paraffin in a heat shaker at 58°C and 400 rev/min overnight. Samples where then embedded in paraffin using a tissue embedding station and small square mounting blocks.

Paraffin blocks were sectioned to 4 µm using Leica HistoCore Arcadia H microtome (Mannheim, Germany). Paraffin sections were stained with standard H&E staining and mounted on glass slides using DePeX as a mounting medium (SERVA, no. 18243.02). The slides were scanned using TissueFaxs (TissueGnostics, Germany).

### RNA isolation and quantitative PCR analysis

Larvae were collected (*n*=3 samples with 20 pooled fish/sample for each group). RNA isolation was performed using the RNeasy Mini Kit (Qiagen, no. 74104). cDNA was synthesised from RNA concentration 735 ng/µl using the iScript cDNA Synthesis Kit (Bio-Rad, no. 1708890) followed by quantitative PCR (qPCR) using the SsoAdvanced Universal SYBR Green Supermix to analyse gene expression elastase and trypsin. qPCR was performed using the BIO-RAD CFX96 C1000 Touch Real-Time PCR Detection System (USA) with the listed primers (ESM Table 5). qPCR analysis was performed using the $2^{-\triangle\triangle{\mathrm C}_{\mathrm t}}$ method [31]; 18s ribosomal RNA C_t_ values were used for normalisation.

### Whole-mount immunofluorescence staining

Immunofluorescence staining was conducted according to [32]. Zebrafish larvae were fixed in 4% paraformaldehyde (PFA) at 4°C overnight. Post-fixation, larvae were washed using 0.1% vol./vol. Tween in PBS (PBS-T) and dehydrated using a stepwise methanol series (25%, 50%, 75% and 100%). The larvae were stored at −20°C for a maximum of 1 month. For rehydration, larvae underwent a stepwise methanol series (75%, 50%, 25%, 0%) followed by incubation in 10 µg/ml proteinase K in PBS-T for 40 min followed by fixing in 4% PFA for 20 min. The larvae were then washed twice for 5 min using PBS-T and incubated in blocking solution (3% BSA in PBS-T, 0.1% serum) for 2 h. Subsequently, larvae were incubated with primary anti-insulin (1:100; Abcam, AB210560) at 4°C for at least 16 h. After washing ten times with PBS-T, larvae were incubated with secondary donkey anti-rabbit Alexa Fluor 594 (1:250; Invitrogen, A21207) and DAPI (1:1000, Sigma, D8417-10 MG) at 4°C for at least 16 h. After washing, larvae were embedded in 1% low-melting agarose in glass bottom dishes and covered with PBS for imaging using a Leica SP8X. Images were acquired at 1 µm intervals, with zoom set to 3 and proper excitation wavelengths. Images were analysed using plugin cell counter in Fiji.

### Electron microscopy processing

The larvae were fixed in cold 2% paraformaldehyde and 0.2% glutaraldehyde in 0.1 mol/l sodium cacodylate, pH 7.4 for a minimum of 24 h. The zebrafish were post-fixed in 1% osmium tetroxide/1.5% potassium ferrocyanide, followed by dehydration (30%, 50%, 70%, 100% ethanol series and water-free acetone) and flat-embedded in Epon as previously reported [9]. Semi-thin 1 µm sections were cut (UC7 ultramicrotome, Leica Microsystems, Vienna, Austria) and toluidine blue staining was performed to select the islet-containing pancreas region. Subsequent ultrathin (80 nm) sections were cut and placed on formvar-coated copper grids (Electron Microscopy Sciences, Hatfield, PA, USA). Sections were stained with 4% neodymium acetate before imaging [33].

### Electron microscopy acquisition, image processing and nanotomy website

Image data were acquired on a Supra 55 scanning electron microscope (SEM; Zeiss, Oberkochen, Germany) using a scanning transmission EM (STEM) detector at 25 kV with 2.5 nm pixel size and an external scan generator ATLAS 5 (Fibics, Ottawa, ON, Canada) as previously described [9]. After image tile stitching, sample datasets were exported as an html file.

### Cell culture, transfection and treatment

HEK293T (ATCC CRL-11268) cells were cultured in DMEM (Gibco, 31966021) containing 10% FBS (Capricorn, FBS-12A) and penicillin/streptomycin (100 µg/µl; Gibco, P11-010). The ER stress reporter (H2BmTurq2-T2A-xbp1 Venus) and apoptosis reporter (FlipGFP-T2A-mCherry) were cloned into pcDNA3.1. Cells were plated in four-compartment 35/10 mm glass bottom cell view plates (Greiner bio-one) at a cell density of 15 × 10^4^ cells/ml in growth medium containing 2 µg/ml poly-l-lysine (Sigma-Aldrich, P6282). The next day, cells were transfected with lipofectamine 2000 according to manufacturer’s protocol (Invitrogen, 11668030). To induce ER stress, cells were treated with tunicamycin (2 µg/ml; Sigma-Aldrich, T7765) for 6 h. To induce apoptosis, cells were treated with raptinal (10 µmol/l; Sigma-Aldrich, sml1745) for 6 h [34].

### Free glucose measurement

Free glucose levels in zebrafish larvae were measured using a Glucose assay kit (Abcam, ab65333) [35]. In short, ten fish/samples were pooled and homogenised/lysed (four times per condition) after which the glucose levels were measured twice per sample using the manufacture’s colorimetric protocol (absorbance 570 nm; BioTek Synergy plate reader; Agilent).

### Statistical analysis

Statistical analysis was conducted using GraphPad Prism version 10.2.3 for Windows (GraphPad Software, Boston, MA USA; www.graphpad.com). Data represent the median or mean values, with bars on graphs indicating the range from the maximum to the minimum data points unless mentioned otherwise. Data distribution was assessed for each experiment and parametric *t* tests were applied for normally distributed samples, whereas non-parametric tests (Mann–Whitney *U* test) were used for non-normally distributed samples. Paired experimental designs were analysed using appropriate paired tests. A value of *p*<0.05 was considered statistically significant.

## Results

### The NFP–NTR system modulates exocrine damage in zebrafish pancreas

**Fig. 1:**
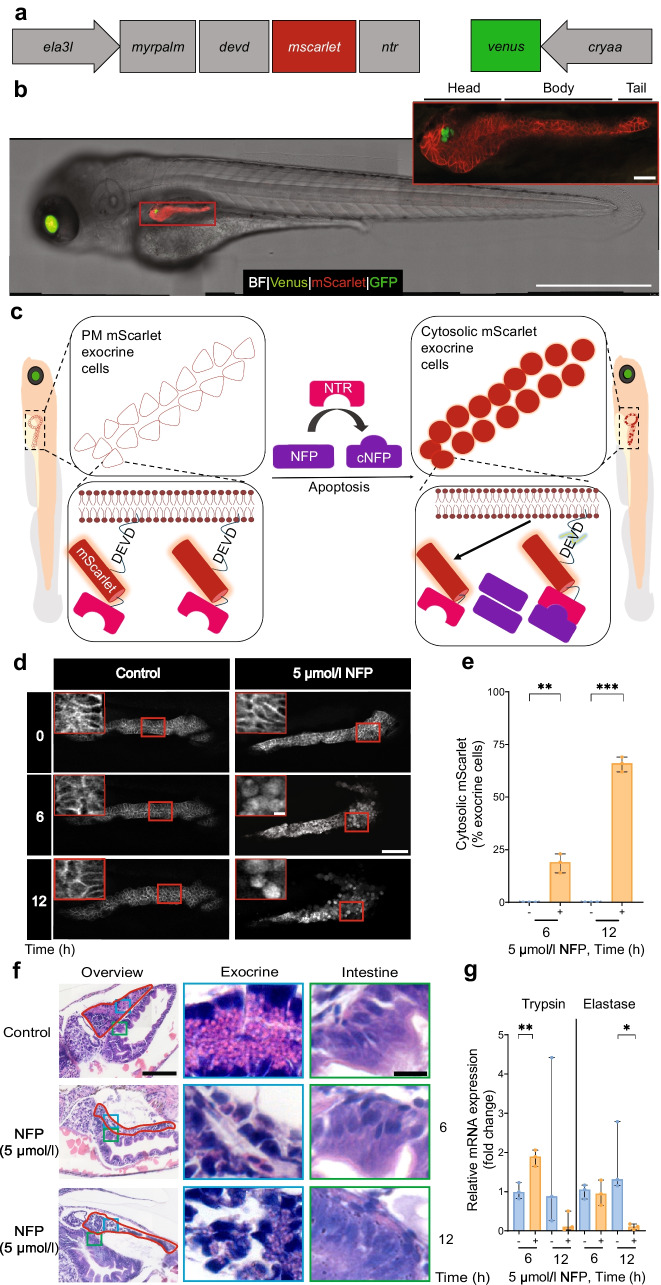
Modulation of exocrine damage in a transgenic zebrafish. (**a**) Schematic of the transgene cloned into the Tol2 plasmid to generate the Tg(*ela3l:myrpalmdevd-mscarlet-ntr;cryaa:venus*) zebrafish, Tg(*ela3l:ntr*). NTR-mScarlet is exclusively targeted to all exocrine plasma membranes through a short myristylation and palmitoylation sequence (*myrpalm*), under control of the elastase A promoter. A *devd* caspase cleavage site sequence is located in between *myrpalm* and *mscarlet-ntr*. Additionally, *venus* under control of the *cryaa* promoter is included for selection of transgenic zebrafish. (**b**) Representative example of the presence of mScarlet^+^ exocrine plasma membrane, Venus^+^ lens and GFP^+^ beta cells in double Tg(*ela3l-ntr;insulin:gfp*) zebrafish larvae at 108 hpf. Scale bar, 1 mm. The magnified image shows mScarlet signal in the three different pancreas portions: head, body and tail, surrounding GFP^+^ beta cells. Scale bar, 50 µm. (**c**) The genetic/chemical NTR–NFP modulation approach. After NFP administration, NTR enzyme transforms NFP into the cytotoxic agent cNFP, leading to apoptosis and caspase activation shown by the translocation of mScarlet from the exocrine plasma membrane to the cytosol. (**d**) Confocal images of mScarlet signal, showing the translocation of mScarlet after 5 µmol/l NFP on NTR^+^ exocrine cells over time; control: 0 µmol/l NFP in 0.1% DMSO. Scale bar, 50 μm or 5 µm (magnified image). (**e**) Cytosolic mScarlet^+^ exocrine cell percentage in response to the induced exocrine damage caused by 5 µmol/l NFP treatment (*n*=3 each). (**f**) H&E staining of larvae sections showing morphological changes of exocrine cells post-damage. Red lines indicate the pancreas, blue boxes indicate the exocrine regions and green boxes indicate the intestinal regions as control (*n*=6 per group). Scale bar, 100 µm or 10 µm (magnified image). (**g**) qPCR analysis of pooled samples of zebrafish larvae showing normalised relative mRNA expression levels of elastase and trypsin with 5 µmol/l NFP (*n*=3). Data represent the median values with bars indicating the range from the maximum to the minimum data points. Unpaired *t* test was used for statistical differences between groups. *p<0.05, **p<0.01, ***p<0.001. Fig. 1c is created in BioRender. Faraj, N. (2025) https://BioRender.com/d22a265. PM, plasma membrane

We used a genetic chemical ablation approach to specifically target exocrine cells and investigate the subsequent effects on beta cells. The generated zebrafish model Tg(*ela3l:ntr*) allows expression of the nitroreductase-encoding gene (*ntr*) and the apoptotic reporter construct (e.g. myristoylation and palmitoylation [*myrpalm*] *devd*-*mscarlet*) specifically in the exocrine pancreas (Fig. 1a). In double Tg(*ela3l:ntr*;*insulin:gfp*) larvae, co-expression of *gfp* under the regulation of the *insulin* promoter allows both the exocrine and beta cells to be investigated. Notably, *ntr* construct expression is not visible in GFP-positive beta cells, indicating no detectable leakage into beta cells (Fig. 1b). In the NFP–NTR system, the exogenous prodrug NFP can be converted by NTR into the cytotoxic agent cNFP. Upon apoptosis, membrane targeting is lost as caspase activity cleaves the DEVD sequence between the myristoylation and palmitoylation (myrpalm) signal and mScarlet (Fig. 1c). The NFP–NTR system remarkably initiated exocrine damage after 6 h of NFP treatment, causing significant damage after 12 h (Fig. 1d, e and ESM Video 2 [available via http://www.nanotomy.org/OA/Faraj2025Diabetologia/]). Heterogeneity and diverse apoptosis features, such as membrane blebbing, were observed mainly in the pancreas head (ESM Fig. 1a). NFP–NTR-mediated damage caused partial or complete disruption of exocrine cells compared with the intestinal region (Fig. 1f). The resulting damage also impaired exocrine cell function, evidenced by decreased elastase expression and probably a reduced trypsin expression after 12 h of 5 µmol/l NFP treatment, while trypsin expression was significantly increased after 6 h (Fig. 1g).

During apoptosis, caspases play a crucial role by cleaving DEVD sites, leading to the exposure of phosphatidylserine (PS) on the outer cell membrane, allowing Annexin V to bind [36]. The effectiveness of the NFP–NTR modulation system in inducing apoptosis in exocrine cells is concluded by the translocation of mScarlet and the binding of Annexin V to PS on the Venus-labelled plasma membrane (ESM Fig. 1b). Overall, we demonstrated that the exocrine damage was efficiently induced by NFP in the Tg(*ela3l:ntr*) zebrafish model.

Moreover, NFP cytotoxicity depended on the presence of NTR, as indicated by mScarlet translocation occurring only when NTR-positive zebrafish were exposed to NFP (ESM Fig. 2a, b). Consequently, NFP cytotoxicity is dependent on the presence of NTR in the Tg(*ela3l:ntr*) model.

### Exocrine damage leads to pancreatic abnormalities and reduced volume in zebrafish

To address generic toxic effects due to NFP administration, Tg(*ela3l:ntr*) larvae were exposed to increasing doses of NFP. While 50 µmol/l NFP was lethal at 3 h, no side effect or toxicity was found after exposure to 10 µmol/l for up to 12 h (ESM Fig. 3a).

**Fig. 2:**
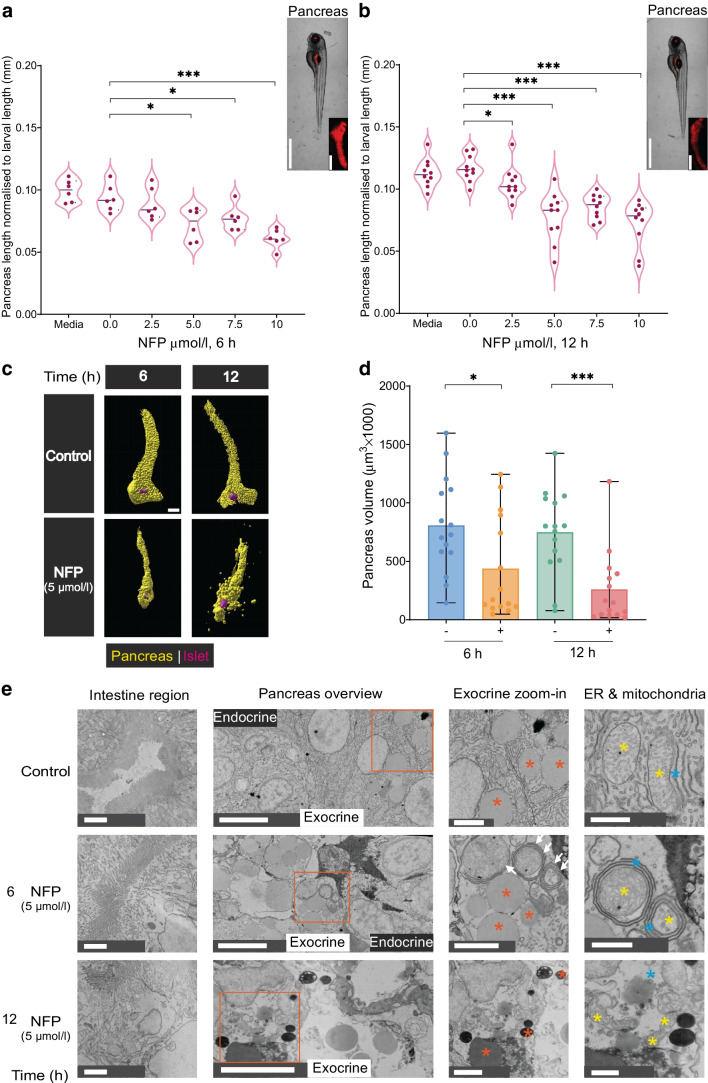
Exocrine damage induces morphological changes and reduces pancreas volume in zebrafish. (**a**, **b**) The pancreas length was measured and normalised to the whole larval length using mScarlet signal after different NFP concentrations and exposure times of 6 h (**a**) and 12 h (**b**), along with media and control (0 µmol/l NFP in 0.1% DMSO) samples (*n*=6 or *n*=10, respectively). Scale bar, 1 mm or 50 µm (magnified image). Unpaired *t* test was used for statistical differences between groups: *p<0.05, ***p<0.001. (**c**) The impact of 5 µmol/l NFP on pancreas volume in the double Tg(*ela3l:ntr;insulin:gfp*) line. Yellow represents mScarlet signal in pancreas; magenta represents the islet portion. Scale bar, 50 µm. (**d**) Bar plots showing the changes in pancreas volume (µm^3^) post-NFP treatment (*n*=15 each). Data represent the mean values with bars indicating the range from the maximum to the minimum data points. Mann–Whitney *U* test was used for statistical differences between groups: *p<0.05, ***p<0.001. (**e**) Electron microscopy images showing ultrastructural alterations within the intestine and pancreas region; the exocrine magnification (zoom-in) and ER and mitochondria images indicate different cellular structural abnormalities (granules, ER whorls, distorted mitochondria) in Tg(*ela3l:ntr*). Orange asterisks indicate exocrine granules; blue asterisks indicate ER; white arrows indicate ER whorls; yellow asterisks indicate mitochondria. Scale bar, 2 µm (intestine and exocrine zoom-in), 5 µm (pancreas overview) and 1 µm (ER and mitochondria). Full electron microscopy data are available via http://www.nanotomy.org/OA/Faraj2025Diabetologia/ and at the BioImage Archive (S-BIAD1479, DOI: https://doi.org/10.6019/S-BIAD1479)

Pancreatic length and volume provide insights into its structural status in relation to pancreatic diseases. Exocrine damage significantly reduced pancreas length (Fig. 2a, b and ESM Fig. 4a–c) and led to a notable decrease in pancreas volume (~ 50%) compared with controls in Tg(*ela3l:ntr*) (Fig. 2c, d and ESM Fig. 3b, ESM Video 2 [available via http://www.nanotomy.org/OA/Faraj2025Diabetologia/]. Cellular stress correlates with alteration in the ER network and mitochondrial morphology. Histological and ultrastructural analysis revealed ER whorls and distorted mitochondria with deformed cristae and therefore suggested an increase in ER–mitochondria contacts in the exocrine compartment (Fig. 2e). These structural alterations are characteristic of ER stress and oxidative stress [37–39]. Furthermore, exocrine granules were seemingly reduced and appeared more compact in NFP-treated Tg(*ela3l:ntr*) larvae, although the islet remained recognisable. In contrast, extensive exocrine damage rendered the islet unrecognisable after 12 h of treatment, while the intestinal region remained structurally unaffected. Apoptotic exocrine cells were observed at 12 h post-NFP treatment, with signs of cellular stress appearing after 6 h (Fig. 2e). Adjacent to damaged exocrine cells, ductal cells in NFP-treated larvae appeared morphologically unchanged (ESM Fig. 3c). Additionally, NFP treatment had no impact on NTR-negative exocrine cells in Tg(*insulin:gfp*) or wild-type AB lines (ESM Fig. 3d). Thus, NFP treatment effectively induced exocrine damage in the Tg(*ela3l:ntr*) model, reducing pancreas length and volume, and ultrastructural analysis revealed that exocrine cells exhibit signs of stress and the islet portion remained identifiable.

### Exocrine damage causes beta cell stress in zebrafish

**Fig. 3:**
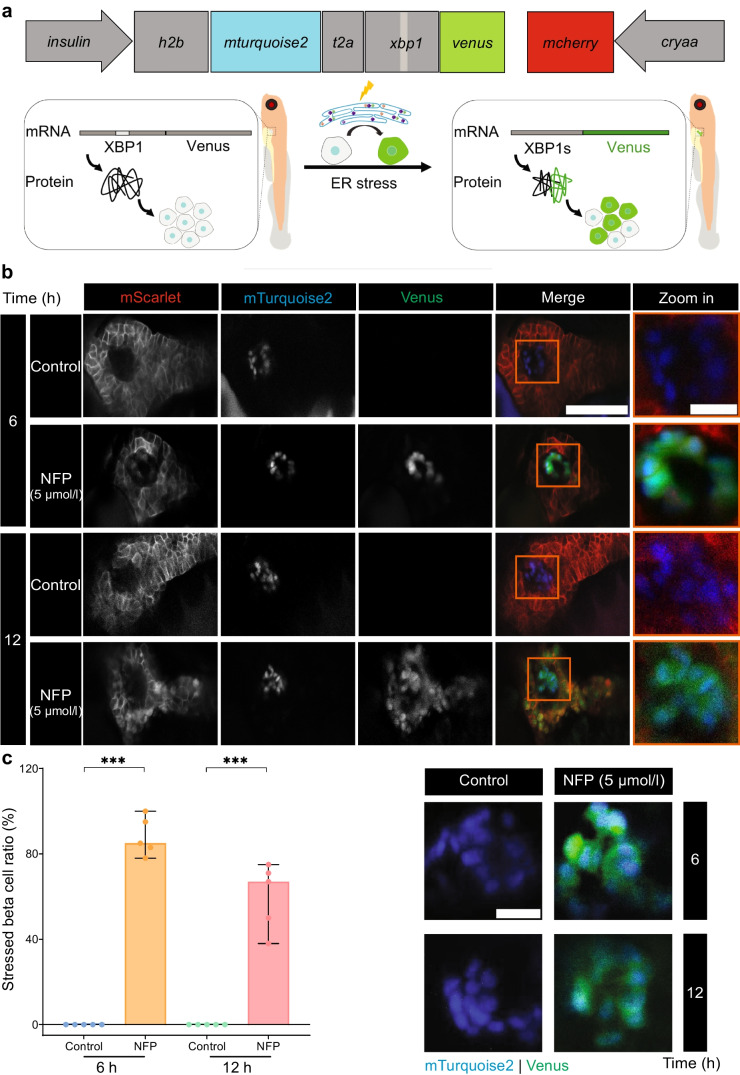
Exocrine damage induces ER stress in beta cells. (**a**) Schematic showing the ER stress reporter under the beta cell-specific insulin promoter; *venus* is fused with *xbp1*, which under normal conditions remains unspliced, resulting in only mTurquoise2^+^ nuclei. Upon ER stress, XBP1s is produced with Venus being in frame and causing stressed beta cells to display mTurquoise2^+^ nuclei and Venus^+^ cytosol in Tg(*insulin:h2b-mturquoise.2-xbp1-venus;cryaa:mcherry*) larvae, briefly Tg(*insulin:xbp1v*). Additionally, *mcherry* under the *cryaa* promoter is included to facilitate the selection of positive transient zebrafish. (**b**) The expression of mTurquoise2 and Venus in beta cells after exocrine damage stimulation by 5 µmol/l NFP. Scale bar, 50 μm or 10 µm (magnified image). (**c**) Quantification of the ratio of stress beta cells to the total number of beta cells after 6 and 12 h of NFP treatment (*n*=5 each). The representative images were used for quantification. Scale bar, 10 µm. Data represent the median values with bars indicating the range from the maximum to the minimum data points. Unpaired *t* test was used for statistical differences between groups. ***p<0.001. Part of Fig. 3a is created in BioRender. Faraj, N. (2025) https://BioRender.com/z06r241

Overactivation of the unfolded protein response (UPR) pathways is an early indicator of beta cell damage [40]. The Tg(*insulin:xbp1v*) reporter line was generated to assess beta cell status following exocrine damage. The generated reporter zebrafish expresses nuclear mTurquoise2 in beta cells and upon ER stress and *xbp1* splicing, with Venus being co-expressed (Fig. 3a). Considering the long development times associated with developing zebrafish lines, the reporter’s activity was first validated in HEK293T cells after chemically induced ER stress. In these cells, the Venus signal is clearly induced upon treatment with ER stress inducer tunicamycin (ESM Fig. 5a). After inducing exocrine damage with 5 µmol/l NFP for 6 h and 12 h, 90% and 60% of beta cells, respectively, were double-positive (mTurquoise2 in the nucleus and Venus in the cytosol) relative to the total number of beta cells (Fig. 3b, c). Moreover, double positivity for mTurquoise2 and GFP was observed in beta cells even after 12 h of exposure to a lower concentration of NFP (2.5 µmol/l) (ESM Fig. 6a).

To distinguish between Venus and autofluorescence, the Venus signal was verified by a λ scan (ESM Fig. 6b). Furthermore, Venus was absent in mTurquoise2-positive beta cells with NTR-negative exocrine background (ESM Fig. 6c), indicating that the ER stress markers emerged in beta cells only after exocrine damage even in a milder condition.

### Exocrine damage does not induce apoptosis in beta cells but does influence their count overtime

**Fig. 4:**
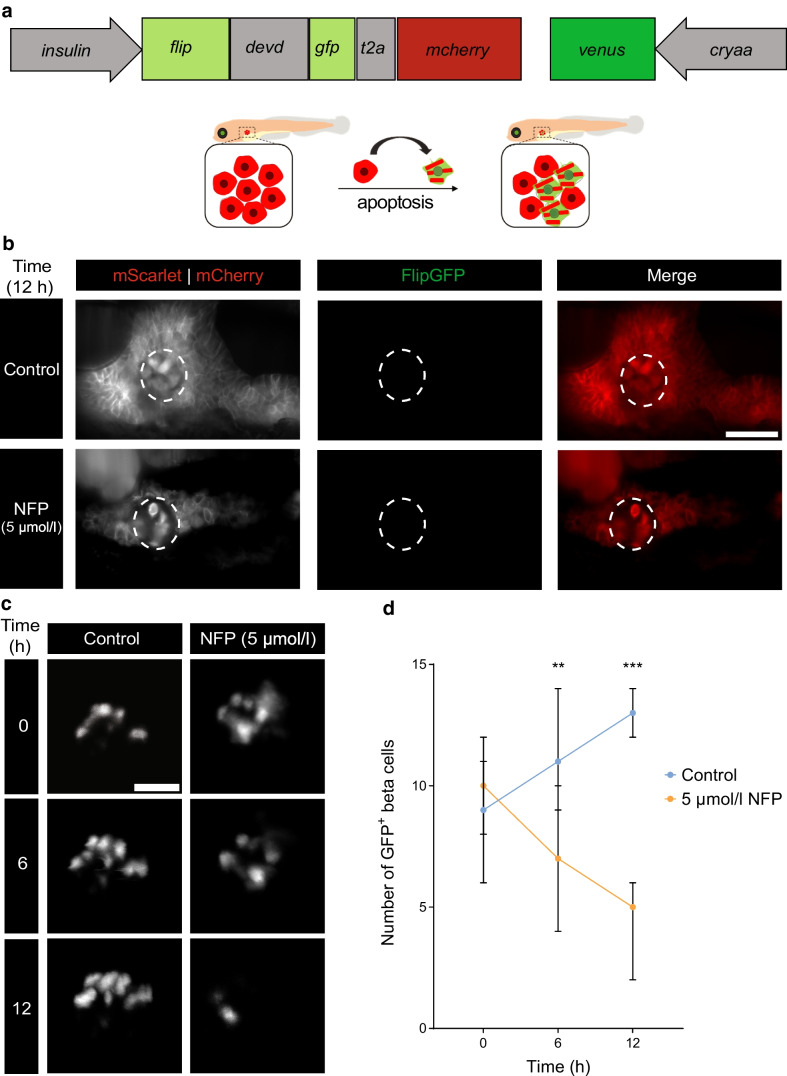
Reduced number of GFP^+^ beta cells with absence of apoptosis post-exocrine damage. (**a**) Apoptosis reporter under the beta cell-specific *insulin* promoter mCherry labels the cytosol of all beta cells. A DEVD caspase cleavage site sequence separates the two β sheets of GFP from each other, resulting in GFP signal loss under normal conditions. Upon apoptosis activation, cleavage of DEVD induces a conformational change within GFP that results in high GFP brightness. (**b**) Light sheet fluorescence images show the presence of mCherry within beta cells without GFP signal after exocrine damage and mScarlet displacement. Scale bar, 50 µm. (**c**) Confocal fluorescence imaging displaying GFP^+^ beta cells within the pancreas over time following NFP treatment. Scale bar, 25 µm. (**d**) Quantification of GFP^+^ beta cells in Tg(*ela3l:ntr*;*insulin:gfp*) larvae at 0, 6 and 12 h after treatment with 5 µmol/l NFP or under control (0 µmol/l NFP in 0.1% DMSO) conditions (*n*=5). Scale bar, 25 µm. Data represent the median values with bars indicating the range from the maximum to the minimum data points. Unpaired multiple *t* tests were used for statistical differences between groups. **p<0.01, ***p<0.001. Part of Fig. 4a is created in BioRender. Faraj, N. (2025) https://BioRender.com/l19l254

Apoptotic beta cells are central to type 1 diabetes onset and apoptosis moreover can be triggered by ER stress [41]. An *insulin:flipgfp-mcherry* reporter zebrafish line was developed, allowing beta cell apoptosis to be detected due to a conformational change of GFP following DEVD cleavage, rendering it fluorescent (Fig. 4a). The validation of the reporter under control of the cytomegalovirus promoter was demonstrated in HEK293 T cells after raptinal treatment to induce apoptosis (ESM Fig. 7a). However, exocrine damage did not lead to beta cell apoptosis in zebrafish, as confirmed by the absence of GFP and the presence of mCherry in beta cells (Fig. 4b and ESM Video 3 [available via http://www.nanotomy.org/OA/Faraj2025Diabetologia/].

Double Tg(*ela3l:ntr*;*insulin:gfp*) zebrafish were generated to determine whether the damage to exocrine cells affects beta cell numbers. In NFP-treated larvae GFP-positive beta cells were present but the number was reduced over time when compared with mock-treated larvae (Fig. 4c, d). Thus, while beta cells did not undergo apoptosis, their numbers decreased following exocrine damage.

### Reduction in islet volume and beta cell function following exocrine damage

**Fig. 5:**
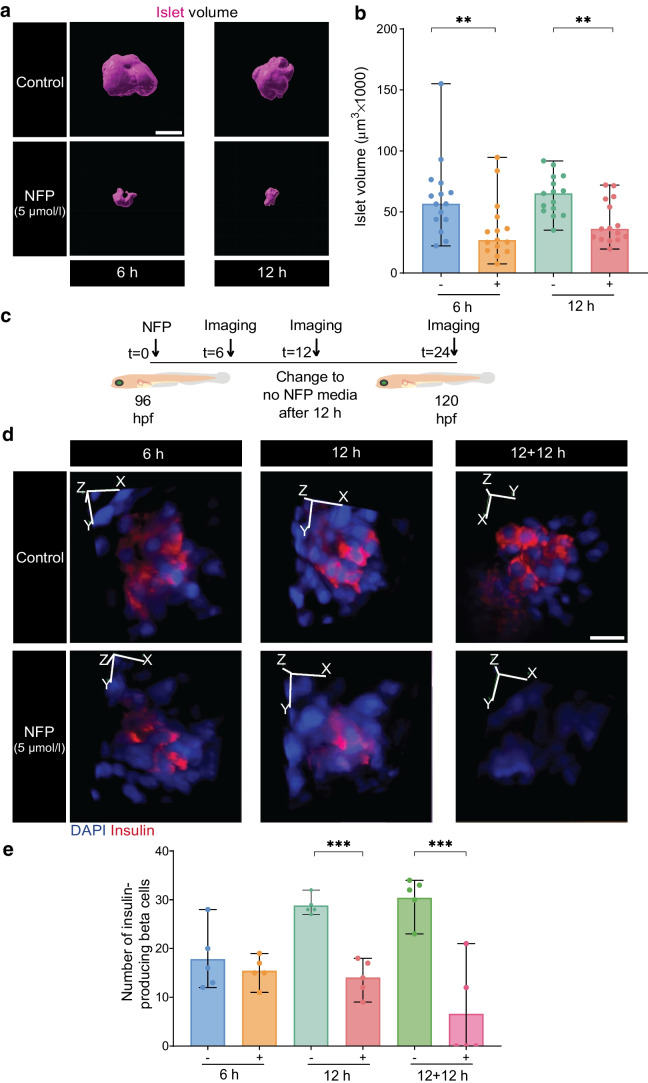
Reduced islet volume and decreased numbers of insulin-producing beta cells following exocrine damage. (**a**) 3D reconstruction of GFP^+^ beta cells and the islet volume measurement after 5 µmol/l NFP treatment. Scale bar, 25 µm. (**b**) Bar blots demonstrating the impact of NFP on the islet volume in Tg(*ela3l:ntr*;*insulin:gfp*) larvae (*n*=15 each). Data represent the mean values with bars indicating the range from the maximum to the minimum data points. Mann–Whitney *U* test was used for statistical differences between groups: **p<0.01. (**c**) Experimental schematic for imaging insulin-labelled Tg(*ela3l:ntr*;*insulin:gfp*) at 96 hpf after 6 and 12 h of NFP treatment followed by 12 h of medium without NFP (recovery time). (**d**, **e**) 3D reconstruction of insulin immunofluorescent staining and DAPI staining at 120 hpf in whole larvae following NFP treatment (*n*=5). Scale bar, 100 µm. Data represent the median values with bars indicating the range from the maximum to the minimum data points. Unpaired *t* test was used for statistical differences between groups:, ***p<0.001. Fig. 5c is created in BioRender. Faraj, N. (2025) https://BioRender.com/g20u364

To investigate whether reduced pancreas volume leads to beta cell atrophy, islet volume was measured. NFP-treated Tg(*ela3l:ntr*;*insulin:gfp*) larvae showed a notable reduction in islet volume (Fig. 5a, b and ESM Fig. 8a). In contrast, the islet volume in Tg(*insulin:gfp*) larvae, which lack NTR in their exocrine cells, remained unchanged after NFP treatment (ESM Fig. 8b). Since the primary islet contains multiple endocrine cells, beta cell count is essential to determine whether changes in beta cell number contribute to the observed reduction in islet volume. The results showed that exocrine damage led to a reduction in insulin-positive beta cell number (~ 50%), both after 12 h of NFP treatment and following a subsequent 12 h recovery period in NFP-free medium (12 + 12 h condition) (Fig. 5c–e and ESM Fig. 8c). Moreover, glucose levels were significantly higher in 96 hpf larvae treated with NFP for 12 h compared with control untreated larvae (ESM Fig. 8d). Therefore, the decrease in islet volume following exocrine damage is associated with a reduction in beta cell number and dysfunction.

## Discussion

Our study reveals that exocrine damage induces ER stress in beta cells and reduces their number in a dynamic zebrafish model. These findings highlight the important role that exocrine damage can play in beta cell impairment. The NTR–NFP system effectively induces exocrine damage within 12 h following NFP exposure, and damaged exocrine cells remain detectable throughout the 24 h NFP treatment period. Interestingly, beta cell recovery in zebrafish typically requires between 24 h and 36 h post-NFP or metronidazole washout [42], suggesting that the recovery process extends beyond the treatment timeframe of this study.

Elevated ER stress triggers beta cell dysfunction, as evidenced in prediabetic NOD mice and donors with type 1 diabetes [43, 44], with ER stress-related genes involved in beta cell malfunction [40]. Our findings indicate that both severe and mild exocrine damage provoke significant ER stress in beta cells, which is associated with a progressive decline in beta cell number and function over time. Additionally, stress signals were observed in exocrine cells following NFP treatment and the impaired ER protein processing in these cells likely contributes to the reduced expression of trypsin and elastase. Previous studies defined that elastase inhibition diminishes beta cell proliferation [45] while protease overexpression promotes it, although the mechanisms remain undefined [46]. Collectively, the observed findings suggest that the decrease in beta cell numbers following exocrine damage may be driven by disrupted proliferative pathways and increased ER stress, which together contribute to beta cell dysfunction, as evidenced by elevated glucose levels.

Despite inducing ER stress, exocrine damage did not trigger beta cell apoptosis, as shown by the absence of GFP in Tg(*ela3l:ntr*;*insulin:flipgfp*) larvae at 120 hpf. Alternatively, lack of GFP may be explained by clearance of apoptotic cells. Notably, Tg(*ela3l:ntr*;*insulin:gfp*) larvae exhibited reduced islet volumes and a decline in GFP-positive beta cells over time, without evidence of apoptosis. Given the beta cell proliferative rate at 120 hpf and the t½ of GFP used as beta cell marker in our system (>24 h), the decrease in beta cell number is likely due to impaired proliferation or reduced neogenesis following NFP treatment.

Exocrine damage induces ER stress in beta cells, leading to reduced beta cell number and volume. However, our findings contrast with those of Schmitner et al, who reported no significant changes in beta cell numbers following exocrine ablation in zebrafish [47]. The discrepancy may stem from differences in experimental approaches, such as the prolonged 96 h diphtheria toxin exposure. Elucidating the underlying mechanisms regulating beta cell viability and proliferation in response to pancreatic damage will be the next step to understand the exocrine/endocrine crosstalk.

The exact role of exocrine damage in diabetes remains unclear but here we find a causal relationship between a smaller pancreatic volume and beta cell malfunction after exocrine damage in vivo. The observed outcome indicates a complex interplay between exocrine damage, ER stress and beta cell dysfunction, underscoring the need for further research. Conversely, another study reported impaired insulin secretion despite normal exocrine function in recent-onset type 1 diabetes [48], suggesting that exocrine abnormalities might only affect a subset of individuals at risk of type 1 diabetes.

Preferably, human material is used to study human diseases; as the rationale of this study was based on findings with human donor material [49], that precludes cause–consequence studies as presented here. Limitations of our approach include the extent of damage induced by the NFP–NTR model, which may better reflect diabetes types with more extensive exocrine damage. Additionally, zebrafish larvae older than 120 hpf are considered laboratory animals, restricting the time window for assessing the impact of exocrine damage to 24–36 h. Moreover, our study also did not assess the role of immune activation in beta cell destruction at this stage, as zebrafish at 120 hpf only have innate immunity [50]. Ongoing studies aim to elucidate the role of the innate immune system such as neutrophils and macrophages in exocrine damage using zebrafish lines that express innate immune cell reporters. The NFP–NTR-induced damage mimics the exocrine atrophy seen in individuals at risk of type 1 diabetes or in those with type 1 diabetes to some extent and provides a valuable model for studying the impact of exocrine damage on beta cells in a controlled, dynamic setting. Additionally, the generated model can help identify novel targets initiating beta cell stress. In conclusion, in the transgenic zebrafish larval model presented here, exocrine damage induces beta cell stress and reduces the number of insulin-positive beta cells, suggesting that exocrine damage may compromise insulin availability and contribute to beta cell dysfunction.

## Supplementary Information

Below is the link to the electronic supplementary material.

ESM (PDF 10158 KB)

## Data Availability

Original light microscopy images and raw electron microscopy data are open access via the BioImage Archive (S-BIAD1479, DOI: 10.6019/S-BIAD1479). All raw EM data in zoomable format and the ESM Videos are available via http://www.nanotomy.org/OA/Faraj2025Diabetologia/.

## Abbreviations

cNFP: Cytotoxic nifurpirinol
ER: Endoplasmic reticulum
hpf: Hours post-fertilisation
myrpalm: Myristoylation and palmitoylation
NFP: Nifurpirinol
NTR: Nitroreductase
PBS-T: 0.1% Tween in PBS
PFA: Paraformaldehyde
PS: Phosphatidylserine
qPCR: Quantitative PCR
