# Supplementary material for: Pancreatic exocrine damage induces beta cell stress in zebrafish larvae
Source: Diabetologia. 2025 Apr 28;68(8):1754–68. doi: 10.1007/s00125-025-06432-4 (PMC12245981; doi:10.1007/s00125-025-06432-4)
Supplement: Supplementary file 1 — ESM (PDF 10158 KB) [file 125_2025_6432_MOESM1_ESM.pdf]

Pancreatic exocrine damage induces beta cell stress in zebrafish larvae

Noura Faraj[1], Willem M.H. Hoogaars[1], B.H. Peter Duinkerken[1], Anouk H. G. Wolters[1], Kim Kats[1], Mette C. Dekkers[2], Arnaud Zaldumbide[2], Ben N. G. Giepmans[1*]

[1] University Medical Center Groningen; [2] Leiden University Medical Center, the Netherlands.

Content:

- **ESM Methods**
- **ESM Tables**

ESM Table 1: Transgenic zebrafish lines

ESM Table 2: Double-crossed transgenic zebrafish lines

ESM Table 3: Addgene plasmids that were used to generate the transgenic lines

ESM Table 4: Primers that were used in plasmid cloning

ESM Table 5: QPCR primers

References

- **ESM Figures**

ESM Fig. 1: Heterogeneity of exocrine cell damage.

ESM Fig. 2: NFP toxicity is NTR dependent.

ESM Fig. 3: NFP toxicity and pancreas volume in transgenic zebrafish larvae

ESM Fig. 4: NFP optimization in transgenic zebrafish larvae.

ESM Fig. 5: Validation of ER stress reporter *in vitro*.

ESM Fig. 6: Assessment of ER stress reporter in zebrafish larvae.

ESM Fig. 7: In vitro validation of Flip-GFP reporter.

ESM Fig. 8: Islet volume and insulin immunostaining post-NFP treatment.

- **ESM Videos**

ESM Video 1: 3D volume of zebrafish pancreas at 107 hpf.

ESM Video 2: Dynamic exocrine cell response after damage induction overtime.

ESM Video 3: *In vivo* monitoring of apoptosis in mCherry+ beta cells

ESM Methods

elaA:myrDEVD-mScarlet-NTR; cryaA:Venus

CCAAGCTTTTTCCAGTGTACTTACCCCACTGACAGACTGTTTTGCTTGAATTATGTTAAT

TACTTGGGTAAAACATGTATTCCAAATCTGATAATATACAATAACAAGCTGTAGTTCATA

TTTAAGCCATCTGAAATGTTAATTACTGTGAGCATTTAGTCTTTTACGGAAAACAGATAG

AAATCACGTCATGAAAACAAATAACATTAGGTTACAAGCAGTACAAATTTGCATGGATTA

ATTACCTTCTAGTCAATTTTGAGTTGAGATATTTTCCATGAGATAACAAAACCCTTAAAC

TGATCAGCAGTGTGGTGACGGAACAACAGCAGCACAATAAGAACCTCATGATCTCTCCGG

TCTCCTTTATCTTTGACAGATTTCAGGAACACCCTGTTATCTAGATGAGATTAAAGCATC

TCCATGTGGGATGATGAGTTTTCTGCAGGCCGTTAAGGGTGAATATTCATACATTATCCT

TTGAATGAGGTCATCTGATACAATAGTTCCTGTGAGGATGAATAATGAGTTTTAGGATTA

AAAGCCATAAGTAAACAGTCACACTTTTAAATTCCCAGATTGCAAAGGCTTTGCTTTGAG

CAAGCCATAAAGTTTCTGTAAGGTGTCTTTGTTTAGATCAAGCAATTTTACATAAACAAA

TTTCAAATCCACACACTAAACATCTTTTTAGCATAAAAACATTTTTTAAAAGGGCGACAC

GGTGGCACAGTGGGTAGCACTGTCGCCTTACAACAAGAAGGTCACTGGTGTGTGCCCAGG

CTGAATCAGTTGGCATTTCTGTGTGGAGTTTGCATGTTCTCCCCATGTGCGTAGGTTTCC

TCCGGGTGCTCTGGTTTCCCCCACACTTCAAAAACATGCGCTATAGGTGAATTGAATAAG

CTAAAATAGCCATAGTGTATGTGCGTGAATGCAAGAGTGTATGGTGTTTCCCTGTGCTGG

GTTGCAGCTGGAAGTTATGCGTAAAACATATGCTGGATAAGTTGGCGGTTAATTCTGCTG

TGGCAACCCCTGATTAATAATGGGACTAAGCTGAAAAAAATGAATAAATGAATTAATATT

TTTACAAGAATGTACACACATTTTATGGTTCAATGGTGAGATATCATAAAACACAAGCTG

GGCAATACACGTGTTTTATCATAGGATACTATGTGTGTGTGTGTGTGTGTGTGTGTGTGT

GTGTGTTAAATGTATGAAACACATAAATGTAAATGCACATACTAATATGTGCTCATTAAG

CATTACAATTTTGCTTAATTTTCAGAGCCAAAAAAAGCAACACAGACGTATGTCAATCCT

GCTGCTAACCTCTTTTCTCTGAGAACTCACCATCTGCTGACCTCCTTCATACAAGACCAC

AGGTACATTCAAATGTAAGATTATTCACAGAGAGAAAAATAATTATTCACAAACCCCATA

TGCTATGGGATATATAGTACTAAATTTGGGTCTGTGAGAGCTGTAATATTTTCATTTGTA

TTCAAAAGTGAGAGTGTCTTTAGTGTATTAATAGATTGTTGAATCAAATAAAGCTGATGA

TGTGTGACAACTGCCTGAGGTAAATATTAAAAATAAAAACCCATTTTCTTCCAATGGTTG

ATCTCTTTCTTATGGGAATTCTAAATAGTCTAGAACTGTATGCTTATGCTAATATATAAA

TCAAGAGAACTAGTCAAGTCAGAGGTTTGTGCTTGAAGGCCATGTGAAAGGTTTCAAAGA

AAGCAGCTGTTCAAAGGCTGATAATTGTTTTTCCAGCTATAACAGGTGGAATACTCAGCA

ATATTGTTTAGAATATTGTGTGGTCAATGTGACATCACAGATAGAGAGGGTTTAAAAAGC

AGTTGATGCCAGTGTTGGCTAGCGTTTAAACTTAAGCTTGGTACCGCCACCATGGGCTGC
S * C Q C W L A F K L K L G T A T M G C

ATCAAGAGCAAGCGCAAGGACAACCTGAACGACGACGAGGTGGACTTGGATCCCATGGTG
I K S K R K D N L N D D E V D L D P M V

AGCAAGGGCGAGGCAGTGATCAAGGAGTTCATGCGGTTCAAGGTGCACATGGAGGGCTCC
S K G E A V I K E F M R F K V H M E G S

```
ATGAACGGCCACGAGTTCGAGATCGAAGGCGAGGGCGAGGGCCGCCCCTACGAGGGCACC
 M  N  G  H  E  F  E  I  E  G  E  G  E  G  R  P  Y  E  G  T

CAGACCGCCAAGCTGAAGGTGACCAAGGGTGGCCCCCTGCCCTTCTCCTGGGACATCCTG
 Q  T  A  K  L  K  V  T  K  G  G  P  L  P  F  S  W  D  I  L

TCCCCTCAGTTCATGTACGGCTCCAGGGCCTTCACCAAGCACCCAGCCGACATCCCCGAC
 S  P  Q  F  M  Y  G  S  R  A  F  T  K  H  P  A  D  I  P  D

TACTATAAGCAGTCCTTCCCCGAGGGCTTCAAGTGGGAGCGCGTGATGAACTTCGAGGAC
 Y  Y  K  Q  S  F  P  E  G  F  K  W  E  R  V  M  N  F  E  D

GGCGGCGCCGTGACCGTGACCCAGGACACCTCCCTGGAGGACGGCACCCTGATCTACAAG
 G  G  A  V  T  V  T  Q  D  T  S  L  E  D  G  T  L  I  Y  K

GTGAAGCTCCGCGGCACCAACTTCCCTCCTGACGGCCCCGTAATGCAGAAGAAGACAATG
 V  K  L  R  G  T  N  F  P  P  D  G  P  V  M  Q  K  K  T  M

GGCTGGGAAGCGTCCACCGAGCGGTTGTACCCCGAGGACGGCGTGCTGAAGGGCGACATT
 G  W  E  A  S  T  E  R  L  Y  P  E  D  G  V  L  K  G  D  I

AAGATGGCCCTGCGCCTGAAGGACGGAGGCCGCTACCTGGCGGACTTCAAGACCACCTAC
 K  M  A  L  R  L  K  D  G  G  R  Y  L  A  D  F  K  T  T  Y

AAGGCCAAGAAGCCCGTGCAGATGCCCGGCGCCTACAACGTCGACCGCAAGTTGGACATC
 K  A  K  K  P  V  Q  M  P  G  A  Y  N  V  D  R  K  L  D  I

ACCTCCCACAACGAGGACTACACCGTGGTGGAACAGTACGAACGCTCCGAGGGCCGCCAC
 T  S  H  N  E  D  Y  T  V  V  E  Q  Y  E  R  S  E  G  R  H

TCCACCGGCGGCATGGACGAGCTGTACAAGGCGGCCGCTATGGACATCATCTCTGTGGCT
 S  T  G  G  M  D  E  L  Y  K           M  D  I  I  S  V  A

CTGAAGAGACATAGCACAAAGGCTTTTGATGCCAGCAAGAAACTGACCCCTGAGCAGGCT
 L  K  R  H  S  T  K  A  F  D  A  S  K  K  L  T  P  E  Q  A

GAGCAGATCAAGACACTGCTGCAGTACAGCCCAAGCAGCCAGAACAGCCAGCCTTGGCAT
 E  Q  I  K  T  L  L  Q  Y  S  P  S  S  Q  N  S  Q  P  W  H

TTTATTGTGGCTTCTACAGAGGAGGGAAAGGCTAGAGTGGCTAAGTCTGCTGCTGGAAAT
 F  I  V  A  S  T  E  E  G  K  A  R  V  A  K  S  A  A  G  N

TATGTGTTCTCTGAGAGAAAGATGCTGGATGCCTCTCATGTGGTGGTGTTCTGTGCTAAG
 Y  V  F  S  E  R  K  M  L  D  A  S  H  V  V  V  F  C  A  K
```

ACAGCCATGGATGATGTGTGGCTGAAACTGGTGGTGGACCAGGAGGATGCTGATGGAAGA
T A M D D V W L K L V V D Q E D A D G R

TTTGCCACACCTGAGGCTAAGGCTGCTAATGATAAGGGAAGAAAGTTCACAGCTGATATG
F A T P E A K A A N D K G R K F T A D M

CACAGAAAGGACCTGCATGATGATGCTGAGTGGATGGCTAAGCAGGTGTATCTGAATGTG
H R K D L H D D A E W M A K Q V Y L N V

GGAAACTTCCTGCTGGGAGTGGCTGCTCTGGGACTGGATGCTGTGCCTATTGAGGGATTT
G N F L L G V A A L G L D A V P I E G F

GATGCTGCTATCCTGGATGCTGAGTTTGGACTGAAGGAGAAGGGATACACCAGCCTGGTG
D A A I L D A E F G L K E K G Y T S L V

GTGGTGCCAGTGGGACACCACTCTGTGGAGGATTTTAATGCTACACTGCCTAAGAGCAGA
V V P V G H H S V E D F N A T L P K S R

CTGCCTCAGAACATCACCCTGACAGAGGTGTAA
L P Q N I T L T E V *

```
CTATTAATAGTGTGCATTCAGTGCAGGATGTTATGTATTTATTTTCTGTAGTGCAAACTT

TTTCAAAGAATTTTCCAGTCTAAGCTTTTCAGTCAAGAAAAAAAAAAAAGCGATTGATTAT

CTGTGATATTTAAAAAAATTAGCTCTTAAATCTGGTGAACCAAGTGGCCACAACACATTA

AATACTGACGTTCTACAGCCATGTAGCTTCATCTGGTCTGGTTTGTTTTGGCAGGCACTT

ATTTTCCGGTGATAGTTGGCCACAAGAGTATTGTCTGAGCCATTCAGTGCTAGACTGTCA

TTCTCAGGTCAGAGTCCACCCCGCTGATTCTGCTGACAACACTGTTCCCACCATGAGATA

ATGCCATTCCAGAGAGATCCATTTGTAAGCCCCTCTTTCTGCAGCACAGGTATATAACCA

GGGGTCTGCCTCCACTAAGGCCGGCACACATCATTTGGGGATCTTTGTACTGTACTAGCC

TCTATTAGCCTCCTACTTGCGTTCAGTTCACATCTTTTGTTCATCAGGTGCAATAAGTTA

CAGTAAGGAGTTACCAGGTCTGACAACATGGTGAGCAAGGGCGAGGAGCTGTTCACCGGG
                            M  V  S  K  G  E  E  L  F  T  G

GTGGTGCCCATCCTGGTCGAGCTGGACGGCGACGTAAACGGCCACAAGTTCAGCGTGTCC
 V  V  P  I  L  V  E  L  D  G  D  V  N  G  H  K  F  S  V  S

GGCGAGGGCGAGGGCGATGCCACCTACGGCAAGCTGACCCTGAAGCTGATCTGCACCACC
 G  E  G  E  G  D  A  T  Y  G  K  L  T  L  K  L  I  C  T  T

GGCAAGCTGCCCGTGCCCTGGCCCACCCTCGTGACCACCCTGGGCTACGGCCTGCAGTGC
 G  K  L  P  V  P  W  P  T  L  V  T  T  L  G  Y  G  L  Q  C

TTCGCCCGCTACCCCGACCACATGAAGCAGCACGACTTCTTCAAGTCCGCCATGCCCGAA
 F  A  R  Y  P  D  H  M  K  Q  H  D  F  F  K  S  A  M  P  E

GGCTACGTCCAGGAGCGCACCATCTTCTTCAAGGACGACGGCAACTACAAGACCCGCGCC
 G  Y  V  Q  E  R  T  I  F  F  K  D  D  G  N  Y  K  T  R  A

GAGGTGAAGTTCGAGGGCGACACCCTGGTGAACCGCATCGAGCTGAAGGGCATCGACTTC
 E  V  K  F  E  G  D  T  L  V  N  R  I  E  L  K  G  I  D  F
```

AAGGAGGACGGCAACATCCTGGGGCACAAGCTGGAGTACAACTACAACAGCCACAACGTC
K E D G N I L G H K L E Y N Y N S H N V

TATATCACCGCCGACAAGCAGAAGAACGGCATCAAGGCCAACTTCAAGATCCGCCACAAC
Y I T A D K Q K N G I K A N F K I R H N

ATCGAGGACGGCGGCGTGCAGCTCGCCGACCACTACCAGCAGAACACCCCCATCGGCGAC
I E D G G V Q L A D H Y Q Q N T P I G D

GGCCCCGTGCTGCTGCCCGACAACCACTACCTGAGCTACCAGTCCGCCCTGAGCAAAGAC
G P V L L P D N H Y L S Y Q S A L S K D

CCCAACGAGAAGCGCGATCACATGGTCCTGCTGGAGTTCGTGACCGCCGCCGGGATCACT
P N E K R D H M V L L E F V T A A G I T

CTCGGCATGGACGAGCTGTACAAGTAA
L G M D E L Y K *

elaA:H2B-mScarlet; cryaA:Venus

CCAAGCTTTTTCCAGTGTACTTACCCCACTGACAGACTGTTTTGCTTGAATTATGTTAAT

TACTTGGGTAAAACATGTATTCCAAATCTGATAATATACAATAACAAGCTGTAGTTCATA

TTTAAGCCATCTGAAATGTTAATTACTGTGAGCATTTAGTCTTTTACGGAAAACAGATAG

AAATCACGTCATGAAAACAAATAACATTAGGTTACAAGCAGTACAAATTTGCATGGATTA

ATTACCTTCTAGTCAATTTTGAGTTGAGATATTTTCCATGAGATAACAAAACCCTTAAAC

TGATCAGCAGTGTGGTGACGGAACAACAGCAGCACAATAAGAACCTCATGATCTCTCCGG

TCTCCTTTATCTTTGACAGATTTCAGGAACACCCTGTTATCTAGATGAGATTAAAGCATC

TCCATGTGGGATGATGAGTTTTCTGCAGGCCGTTAAGGGTGAATATTCATACATTATCCT

TTGAATGAGGTCATCTGATACAATAGTTCCTGTGAGGATGAATAATGAGTTTTAGGATTA

AAAGCCATAAGTAAACAGTCACACTTTTAAATTCCCAGATTGCAAAGGCTTTGCTTTGAG

CAAGCCATAAAGTTTCTGTAAGGTGTCTTTGTTTAGATCAAGCAATTTTACATAAACAAA

TTTCAAATCCACACACTAAACATCTTTTTAGCATAAAAACATTTTTTAAAAGGGCGACAC

GGTGGCACAGTGGGTAGCACTGTCGCCTTACAACAAGAAGGTCACTGGTGTGTGCCCAGG

CTGAATCAGTTGGCATTTCTGTGTGGAGTTTGCATGTTCTCCCCATGTGCGTAGGTTTCC

TCCGGGTGCTCTGGTTTCCCCCACACTTCAAAAACATGCGCTATAGGTGAATTGAATAAG

CTAAAATAGCCATAGTGTATGTGCGTGAATGCAAGAGTGTATGGTGTTTCCCTGTGCTGG

GTTGCAGCTGGAAGTTATGCGTAAAACATATGCTGGATAAGTTGGCGGTTAATTCTGCTG

TGGCAACCCCTGATTAATAATGGGACTAAGCTGAAAAAAATGAATAAATGAATTAATATT

TTTACAAGAATGTACACACATTTTATGGTTCAATGGTGAGATATCATAAAACACAAGCTG

GGCAATACACGTGTTTTATCATAGGATACTATGTGTGTGTGTGTGTGTGTGTGTGTGTGT

GTGTGTTAAATGTATGAAACACATAAATGTAAATGCACATACTAATATGTGCTCATTAAG

CATTACAATTTTGCTTAATTTTCAGAGCCAAAAAAAGCAACACAGACGTATGTCAATCCT

GCTGCTAACCTCTTTTCTCTGAGAACTCACCATCTGCTGACCTCCTTCATACAAGACCAC

AGGTACATTCAAATGTAAGATTATTCACAGAGAGAAAAATAATTATTCACAAACCCCATA

TGCTATGGGATATATAGTACTAAATTTGGGTCTGTGAGAGCTGTAATATTTTCATTTGTA

TTCAAAAGTGAGAGTGTCTTTAGTGTATTAATAGATTGTTGAATCAAATAAAGCTGATGA

TGTGTGACAACTGCCTGAGGTAAATATTAAAAATAAAAACCCATTTTCTTCCAATGGTTG

ATCTCTTTCTTATGGGAATTCTAAATAGTCTAGAACTGTATGCTTATGCTAATATATAAA

TCAAGAGAACTAGTCAAGTCAGAGGTTTGTGCTTGAAGGCCATGTGAAAGGTTTCAAAGA

AAGCAGCTGTTCAAAGGCTGATAATTGTTTTTCCAGCTATAACAGGTGGAATACTCAGCA

ATATTGTTTAGAATATTGTGTGGTCAATGTGACATCACAGATAGAGAGGGTTTAAAAAGC

AGTTGATGCCAGTGTTGGCTAGCGTTTAAACTTAAGCTTGGTACCGCCACCATGCCAGAG
M P E

CCAGCGAAGTCTGCTCCCGCCCCGAAAAAGGGCTCCAAGAAGGCGGTGACTAAGGCGCAG
P A K S A P A P K K G S K K A V T K A Q

AAGAAAGGCGGCAAGAAGCGCAAGCGCAGCCGCAAGGAGAGCTATTCCATCTATGTGTAC
K K G G K K R K R S R K E S Y S I Y V Y

AAGGTTCTGAAGCAGGTCCACCCTGACACCGGCATTTCGTCCAAGGCCATGGGCATCATG
K V L K Q V H P D T G I S S K A M G I M

AATTCGTTTGTGAACGACATTTTCGAGCGCATCGCAGGTGAGGCTTCCCGCCTGGCGCAT
N S F V N D I F E R I A G E A S R L A H

TACAACAAGCGCTCGACCATCACCTCCAGGGAGATCCAGACGGCCGTGCGCCTGCTGCTG
Y N K R S T I T S R E I Q T A V R L L L

CCTGGGGAGTTGGCCAAGCACGCCGTGTCCGAGGGTACTAAGGCCATCACCAAGTACACC
P G E L A K H A V S E G T K A I T K Y T

AGCGCTAAGGATCCCATGGTGAGCAAGGGCGAGGCAGTGATCAAGGAGTTCATGCGGTTC
S A K M V S K G E A V I K E F M R F

AAGGTGCACATGGAGGGCTCCATGAACGGCCACGAGTTCGAGATCGAAGGCGAGGGCGAG
K V H M E G S M N G H E F E I E G E G E

GGCCGCCCCTACGAGGGCACCCAGACCGCCAAGCTGAAGGTGACCAAGGGTGGCCCCCTG
G R P Y E G T Q T A K L K V T K G G P L

CCCTTCTCCTGGGACATCCTGTCCCCTCAGTTCATGTACGGCTCCAGGGCCTTCACCAAG
P F S W D I L S P Q F M Y G S R A F T K

CACCCAGCCGACATCCCCGACTACTATAAGCAGTCCTTCCCCGAGGGCTTCAAGTGGGAG
H P A D I P D Y Y K Q S F P E G F K W E

CGCGTGATGAACTTCGAGGACGGCGGCGCCGTGACCGTGACCCAGGACACCTCCCTGGAG
R V M N F E D G G A V T V T Q D T S L E

GACGGCACCCTGATCTACAAGGTGAAGCTCCGCGGCACCAACTTCCCTCCTGACGGCCCC
D G T L I Y K V K L R G T N F P P D G P

GTAATGCAGAAGAAGACAATGGGCTGGGAAGCGTCCACCGAGCGGTTGTACCCCGAGGAC
V M Q K K T M G W E A S T E R L Y P E D

GGCGTGCTGAAGGGCGACATTAAGATGGCCCTGCGCCTGAAGGACGGAGGCCGCTACCTG
G V L K G D I K M A L R L K D G G R Y L

GCGGACTTCAAGACCACCTACAAGGCCAAGAAGCCCGTGCAGATGCCCGGCGCCTACAAC
A D F K T T Y K A K K P V Q M P G A Y N

GTCGACCGCAAGTTGGACATCACCTCCCACAACGAGGACTACACCGTGGTGGAACAGTAC
V D R K L D I T S H N E D Y T V V E Q Y

GAACGCTCCGAGGGCCGCCACTCCACCGGCGGCATGGACGAGCTGTACAAGTAA
E R S E G R H S T G G M D E L Y K *

```
CTATTAATAGTGTGCATTCAGTGCAGGATGTTATGTATTTATTTTCTGTAGTGCAAACTT

TTTCAAAGAATTTTCCAGTCTAAGCTTTTCAGTCAAGAAAAAAAAAAAAGCGATTGATTAT

CTGTGATATTTAAAAAAATTAGCTCTTAAATCTGGTGAACCAAGTGGCCACAACACATTA

AATACTGACGTTCTACAGCCATGTAGCTTCATCTGGTCTGGTTTGTTTTGGCAGGCACTT

ATTTTCCGGTGATAGTTGGCCACAAGAGTATTGTCTGAGCCATTCAGTGCTAGACTGTCA

TTCTCAGGTCAGAGTCCACCCCGCTGATTCTGCTGACAACACTGTTCCCACCATGAGATA

ATGCCATTCCAGAGAGATCCATTTGTAAGCCCCTCTTTCTGCAGCACAGGTATATAACCA

GGGGTCTGCCTCCACTAAGGCCGGCACACATCATTTGGGGATCTTTGTACTGTACTAGCC

TCTATTAGCCTCCTACTTGCGTTCAGTTCACATCTTTTGTTCATCAGGTGCAATAAGTTA

CAGTAAGGAGTTACCAGGTCTGACAACATGGTGAGCAAGGGCGAGGAGCTGTTCACCGGG
                            M  V  S  K  G  E  E  L  F  T  G

GTGGTGCCCATCCTGGTCGAGCTGGACGGCGACGTAAACGGCCACAAGTTCAGCGTGTCC
 V  V  P  I  L  V  E  L  D  G  D  V  N  G  H  K  F  S  V  S

GGCGAGGGCGAGGGCGATGCCACCTACGGCAAGCTGACCCTGAAGCTGATCTGCACCACC
 G  E  G  E  G  D  A  T  Y  G  K  L  T  L  K  L  I  C  T  T

GGCAAGCTGCCCGTGCCCTGGCCCACCCTCGTGACCACCCTGGGCTACGGCCTGCAGTGC
 G  K  L  P  V  P  W  P  T  L  V  T  T  L  G  Y  G  L  Q  C

TTCGCCCGCTACCCCGACCACATGAAGCAGCACGACTTCTTCAAGTCCGCCATGCCCGAA
 F  A  R  Y  P  D  H  M  K  Q  H  D  F  F  K  S  A  M  P  E

GGCTACGTCCAGGAGCGCACCATCTTCTTCAAGGACGACGGCAACTACAAGACCCGCGCC
 G  Y  V  Q  E  R  T  I  F  F  K  D  D  G  N  Y  K  T  R  A

GAGGTGAAGTTCGAGGGCGACACCCTGGTGAACCGCATCGAGCTGAAGGGCATCGACTTC
 E  V  K  F  E  G  D  T  L  V  N  R  I  E  L  K  G  I  D  F
```

```
AAGGAGGACGGCAACATCCTGGGGCACAAGCTGGAGTACAACTACAACAGCCACAACGTC
 K  E  D  G  N  I  L  G  H  K  L  E  Y  N  Y  N  S  H  N  V

TATATCACCGCCGACAAGCAGAAGAACGGCATCAAGGCCAACTTCAAGATCCGCCACAAC
 Y  I  T  A  D  K  Q  K  N  G  I  K  A  N  F  K  I  R  H  N

ATCGAGGACGGCGGCGTGCAGCTCGCCGACCACTACCAGCAGAACACCCCCATCGGCGAC
 I  E  D  G  G  V  Q  L  A  D  H  Y  Q  Q  N  T  P  I  G  D

GGCCCCGTGCTGCTGCCCGACAACCACTACCTGAGCTACCAGTCCGCCCTGAGCAAAGAC
 G  P  V  L  L  P  D  N  H  Y  L  S  Y  Q  S  A  L  S  K  D

CCCAACGAGAAGCGCGATCACATGGTCCTGCTGGAGTTCGTGACCGCCGCCGGGATCACT
 P  N  E  K  R  D  H  M  V  L  L  E  F  V  T  A  A  G  I  T

CTCGGCATGGACGAGCTGTACAAGTAA
 L  G  M  D  E  L  Y  K  *
```

ins:H2B- mTurquoise2-T2A-XBP1-Venus; cryaA:mCherry

TTCAGCATTGAACTCTATTGTATTATAAATGGGTATACTTATGTTTGTACCTTAATAAGT

TCATTATATAATATTTAAAATGATACTGCATCATTAATTGATATGTTTTCTAGTTGTTTT

TATATATTAGCAACTAAATTAGGAAATTATCCATAGTAAATTTAATGTAATATGATTTGT

TATATTTAACTTCAGCCCACAGTCTAGTTTAGTTGTTGGTCCCTTATGAGAAAAAGTTTG

GGCACCTCTGCTCTAGTGCTTGTAAACATTTTTGAGTATCTATAGTTGAACATGAAAGCA

TTTTTTTGTTTGTTTTTTAAGAAAATGAAGAAAGCTAAAAACCTGTATCCACTGACTTCC

ATTAAAAAAGCAAATACTAGGGATGTCTATGGTGTCTTTTGTGTTCAACAAATAAGCTAA

CAAATAGTAACTGCTGACAGAACATTACATTTTGTGTGAATTCCCTTAAATTGTGTTTAA

AACTGTAGAGAAATCATCTAATAATAAGAAATTATCAAGAAATAAACACAAAATGATCTA

ATAGTATACTATAAAATCTTTCAAAATCCAGGCCACTACCCTATGACTAAAACCACACAT

ATTAGGTTTTAAAAAAAAAGTCACAATGAAGACGTCACAAACGTTCCTGAAGACTAATGAC

TCGTCTAACCCCATCTTACCTTCTGCTGTTTCACTCAAAGAAAAGCCACCAGCTGTCTCT

GTGATCTGCTCATTCTGTGTAAACTTGTGTTCTCAACAATTAACAATTAACCAAAAGCCT

CTGGAGCTGCGTGAGCATCACGATGTGTTTGTCTTCTGTGAGCGTGGCAGTGTCCACGAA

GTCAATGTGAATTGGTTGGCCAAACACAGCTAATGTGGCATGTGTGATTGGACAGCCACT

TAAAAGTGTTAAAGTGTGTTTACGCACAGGATTGGTTTTCCGCTGAATCAGGCAAAACCA

GCAGAACCGATCTAAACTCGAGTATAAAAGTGAGTAGGAGATGCTATTCGTCCCAAAACA

TCTCCACCACCATATCCACCATTCCTCGCCTCTGCTTCGAGAACAGGTGAGTGTCGAGCG

GGATGGTAAATCTACAGAGAATGCGGAGTGTAGCTTGTGTACATGTTTTTGATTAACAGA

GATTGTATGTGTGTGTTTGTGTCAGTGTGA

```
ATGCCAGAGCCAGCGAAGTCTGCTCCCGCCCCGAAAAAGGGCTCCAAGAAGGCGGTGACT
 M  P  E  P  A  K  S  A  P  A  P  K  K  G  S  K  K  A  V  T

AAGGCGCAGAAGAAAGGCGGCAAGAAGCGCAAGCGCAGCCGCAAGGAGAGCTATTCCATC
 K  A  Q  K  K  G  G  K  K  R  K  R  S  R  K  E  S  Y  S  I

TATGTGTACAAGGTTCTGAAGCAGGTCCACCCTGACACCGGCATTTCGTCCAAGGCCATG
 Y  V  Y  K  V  L  K  Q  V  H  P  D  T  G  I  S  S  K  A  M

GGCATCATGAATTCGTTTGTGAACGACATTTTCGAGCGCATCGCAGGTGAGGCTTCCCGC
 G  I  M  N  S  F  V  N  D  I  F  E  R  I  A  G  E  A  S  R

CTGGCGCATTACAACAAGCGCTCGACCATCACCTCCAGGGAGATCCAGACGGCCGTGCGC
 L  A  H  Y  N  K  R  S  T  I  T  S  R  E  I  Q  T  A  V  R

CTGCTGCTGCCTGGGGAGTTGGCCAAGCACGCCGTGTCCGAGGGTACTAAGGCCATCACC
 L  L  L  P  G  E  L  A  K  H  A  V  S  E  G  T  K  A  I  T

AAGTACACCAGCGCTAAGGATCCACCGGTCGCCACCATGGTGAGCAAGGGCGAGGAGCTG
 K  Y  T  S  A  K                    M  V  S  K  G  E  E  L

TTCACCGGGGTGGTGCCCATCCTGGTCGAGCTGGACGGCGACGTAAACGGCCACAAGTTC
 F  T  G  V  V  P  I  L  V  E  L  D  G  D  V  N  G  H  K  F

AGCGTGTCCGGCGAGGGCGAGGGCGATGCCACCTACGGCAAGCTGACCCTGAAGTTCATC
 S  V  S  G  E  G  E  G  D  A  T  Y  G  K  L  T  L  K  F  I

TGCACCACCGGCAAGCTGCCCGTGCCCTGGCCCACCCTCGTGACCACCCTGTCCTGGGGC
 C  T  T  G  K  L  P  V  P  W  P  T  L  V  T  T  L  S  W  G

GTGCAGTGCTTCGCCCGCTACCCCGACCACATGAAGCAGCACGACTTCTTCAAGTCCGCC
 V  Q  C  F  A  R  Y  P  D  H  M  K  Q  H  D  F  F  K  S  A

ATGCCCGAAGGCTACGTCCAGGAGCGCACCATCTTCTTCAAGGACGACGGCAACTACAAG
 M  P  E  G  Y  V  Q  E  R  T  I  F  F  K  D  D  G  N  Y  K

ACCCGCGCCGAGGTGAAGTTCGAGGGCGACACCCTGGTGAACCGCATCGAGCTGAAGGGC
 T  R  A  E  V  K  F  E  G  D  T  L  V  N  R  I  E  L  K  G

ATCGACTTCAAGGAGGACGGCAACATCCTGGGGCACAAGCTGGAGTACAACTACTTTAGC
 I  D  F  K  E  D  G  N  I  L  G  H  K  L  E  Y  N  Y  F  S

GACAACGTCTATATCACCGCCGACAAGCAGAAGAACGGCATCAAGGCCAACTTCAAGATC
 D  N  V  Y  I  T  A  D  K  Q  K  N  G  I  K  A  N  F  K  I

CGCCACAACATCGAGGACGGCGGCGTGCAGCTCGCCGACCACTACCAGCAGAACACCCCC
 R  H  N  I  E  D  G  G  V  Q  L  A  D  H  Y  Q  Q  N  T  P

ATCGGCGACGGCCCCGTGCTGCTGCCCGACAACCACTACCTGAGCACCCAGTCCAAGCTG
 I  G  D  G  P  V  L  L  P  D  N  H  Y  L  S  T  Q  S  K  L

AGCAAAGACCCCAACGAGAAGCGCGATCACATGGTCCTGCTGGAGTTCGTGACCGCCGCC
 S  K  D  P  N  E  K  R  D  H  M  V  L  L  E  F  V  T  A  A

GGGATCACTCTCGGCATGGACGAGCTGTACAAGGAGGGCAGAGGAAGTCTTCTAACATGC
 G  I  T  L  G  M  D  E  L  Y  K  E  G  R  G  S  L  L  T  C

GGTGACGTGGAGGAGAATCCCGGCCCTAAGCTTCTCACCATGGAGCAAAAGCTCATTTCT
 G  D  V  E  E  N  P  G  P

GAAGAGGACTTGAATTCAAGGCTGCTCAGTGAGAATGAGGAGCTGAGACAGAGACTGGGG
                   L  L  S  E  N  E  E  L  R  Q  R  L  G
```

```
TTGGATACCTTGGAAACAAAGGAGCAGGTTCAGGTACTGGAGTCCGCAGTGAGCGATTTA
 L   D   T   L   E   T   K   E   Q   V   Q   V   L   E   S   A   V   S   D   L

GGTTTGGTGACCGGGTCTTCTGAGTCCGCAGCACTCAGGCTACGTGTGCCTCCGCAGCAG
 G   L   V   T   G   S   S   E   S   A   A   L   R   L   R   V   P   P   Q   Q

GTGCAGGCCCAGCAGTCCCCAAATCTGAAGACTTCACCATGGATACTCACAGCCCTGGCC
 V   Q   A   Q   Q   S   P   N   L   K   T   S   P   W   I   L   T   A   L   A

CTGCAGACTCTGAGTCTGATGCGGATCCAGCCACCATGGTGAGCAAGGGCGAGGAGCTGT
 L   Q   T   L   :                   Q   P   P   W   *   A   R   A   R   S   C

TCACCGGGGTGGTGCCCATCCTGGTCGAGCTGGACGGCGACGTAAACGGCCACAAGTTCA
 S   P   G   W   C   P   S   W   S   S   W   T   A   T   *   T   A   T   S   S

GCGTGTCCGGCGAGGGCGAGGGCGATGCCACCTACGGCAAGCTGACCCTGAAGCTGATCT
 A   C   P   A   R   A   R   A   M   P   P   T   A   S   *   P   *   S   *   S

GCACCACCGGCAAGCTGCCCGTGCCCTGGCCCACCCTCGTGACCACCCTGGGCTACGGCC
 A   P   P   A   S   C   P   C   P   G   P   P   S   *   P   P   W   A   T   A

TGCAGTGCTTCGCCCGCTACCCCGACCACATGAAGCAGCACGACTTCTTCAAGTCCGCCA
 C   S   A   S   P   A   T   P   T   T   *   S   S   T   T   S   S   S   P   P

TGCCCGAAGGCTACGTCCAGGAGCGCACCATCTTCTTCAAGGACGACGGCAACTACAAGA
 C   P   K   A   T   S   R   S   A   P   S   S   S   R   T   T   A   T   T   R

CCCGCGCCGAGGTGAAGTTCGAGGGCGACACCCTGGTGAACCGCATCGAGCTGAAGGGCA
 P   A   P   R   *   S   S   R   A   T   P   W   *   T   A   S   S   *   R   A

TCGACTTCAAGGAGGACGGCAACATCCTGGGGCACAAGCTGGAGTACAACTACAACAGCC
 S   T   S   R   R   T   A   T   S   W   G   T   S   W   S   T   T   T   T   A

ACAACGTCTATATCACCGCCGACAAGCAGAAGAACGGCATCAAGGCCAACTTCAAGATCC
 T   T   S   I   S   P   P   T   S   R   R   T   A   S   R   P   T   S   R   S

GCCACAACATCGAGGACGGCGGCGTGCAGCTCGCCGACCACTACCAGCAGAACACCCCCA
 A   T   T   S   R   T   A   A   C   S   S   P   T   T   T   S   R   T   P   P
```

```
CTATTAATAGTGTGCATTCAGTGCAGGATGTTATGTATTTATTTTCTGTAGTGCAAACTT

TTTCAAAGAATTTTCCAGTCTAAGCTTTTCAGTCAAGAAAAAAAAAAAAGCGATTGATTAT

CTGTGATATTTAAAAAAATTAGCTCTTAAATCTGGTGAACCAAGTGGCCACAACACATTA

AATACTGACGTTCTACAGCCATGTAGCTTCATCTGGTCTGGTTTGTTTTGGCAGGCACTT

ATTTTCCGGTGATAGTTGGCCACAAGAGTATTGTCTGAGCCATTCAGTGCTAGACTGTCA

TTCTCAGGTCAGAGTCCACCCCGCTGATTCTGCTGACAACACTGTTCCCACCATGAGATA

ATGCCATTCCAGAGAGATCCATTTGTAAGCCCCTCTTTCTGCAGCACAGGTATATAACCA

GGGGTCTGCCTCCACTAAGGCCGGCACACATCATTTGGGGATCTTTGTACTGTACTAGCC

TCTATTAGCCTCCTACTTGCGTTCAGTTCACATCTTTTGTTCATCAGGTGCAATAAGTTA

CAGTAAGGAGTTACCAGGTCTGACAACATGGTGAGCAAGGGCGAGGAGGATAACATGGCC
                            M  V  S  K  G  E  E  D  N  M  A

ATCATCAAGGAGTTCATGCGCTTCAAGGTGCACATGGAGGGCTCCGTGAACGGCCACGAG
 I  I  K  E  F  M  R  F  K  V  H  M  E  G  S  V  N  G  H  E

TTCGAGATCGAGGGCGAGGGCGAGGGCCGCCCCTACGAGGGCACCCAGACCGCCAAGCTG
 F  E  I  E  G  E  G  E  G  R  P  Y  E  G  T  Q  T  A  K  L

AAGGTGACCAAGGGTGGCCCCCTGCCCTTCGCCTGGGACATCCTGTCCCCTCAGTTCATG
 K  V  T  K  G  G  P  L  P  F  A  W  D  I  L  S  P  Q  F  M

TACGGCTCCAAGGCCTACGTGAAGCACCCCGCCGACATCCCCGACTACTTGAAGCTGTCC
 Y  G  S  K  A  Y  V  K  H  P  A  D  I  P  D  Y  L  K  L  S

TTCCCCGAGGGCTTCAAGTGGGAGCGCGTGATGAACTTCGAGGACGGCGGCGTGGTGACC
 F  P  E  G  F  K  W  E  R  V  M  N  F  E  D  G  G  V  V  T

GTGACCCAGGACTCCTCCCTGCAGGACGGCGAGTTCATCTACAAGGTGAAGCTGCGCGGC
 V  T  Q  D  S  S  L  Q  D  G  E  F  I  Y  K  V  K  L  R  G

ACCAACTTCCCCTCCGACGGCCCCGTAATGCAGAAGAAGACCATGGGCTGGGAGGCCTCC
 T  N  F  P  S  D  G  P  V  M  Q  K  K  T  M  G  W  E  A  S

TCCGAGCGGATGTACCCCGAGGACGGCGCCCTGAAGGGCGAGATCAAGCAGAGGCTGAAG
 S  E  R  M  Y  P  E  D  G  A  L  K  G  E  I  K  Q  R  L  K

CTGAAGGACGGCGGCCACTACGACGCTGAGGTCAAGACCACCTACAAGGCCAAGAAGCCC
 L  K  D  G  G  H  Y  D  A  E  V  K  T  T  Y  K  A  K  K  P

GTGCAGCTGCCCGGCGCCTACAACGTCAACATCAAGTTGGACATCACCTCCCACAACGAG
 V  Q  L  P  G  A  Y  N  V  N  I  K  L  D  I  T  S  H  N  E

GACTACACCATCGTGGAACAGTACGAACGCGCCGAGGGCCGCCACTCCACCGGCGGCATG
 D  Y  T  I  V  E  Q  Y  E  R  A  E  G  R  H  S  T  G  G  M

GACGAGCTGTACAAGTAA
 D  E  L  Y  K  *
```

ins:FlipGFP-T2A- Cherry; cryaA: Venus

TTCAGCATTGAACTCTATTGTATTATAAATGGGTATACTTATGTTTGTACCTTAATAAGT

TCATTATATAATATTTAAAATGATACTGCATCATTAATTGATATGTTTTCTAGTTGTTTT

TATATATTAGCAACTAAATTAGGAAATTATCCATAGTAAATTTAATGTAATATGATTTGT

TATATTTAACTTCAGCCCACAGTCTAGTTTAGTTGTTGGTCCCTTATGAGAAAAAGTTTG

GGCACCTCTGCTCTAGTGCTTGTAAACATTTTTGAGTATCTATAGTTGAACATGAAAGCA

TTTTTTTGTTTGTTTTTTAAGAAAATGAAGAAAGCTAAAAACCTGTATCCACTGACTTCC

ATTAAAAAAGCAAATACTAGGGATGTCTATGGTGTCTTTTGTGTTCAACAAATAAGCTAA

CAAATAGTAACTGCTGACAGAACATTACATTTTGTGTGAATTCCCTTAAATTGTGTTTAA

AACTGTAGAGAAATCATCTAATAATAAGAAATTATCAAGAAATAAACACAAAATGATCTA

ATAGTATACTATAAAATCTTTCAAAATCCAGGCCACTACCCTATGACTAAAACCACACAT

ATTAGGTTTTAAAAAAAAGTCACAATGAAGACGTCACAAACGTTCCTGAAGACTAATGAC

TCGTCTAACCCCATCTTACCTTCTGCTGTTTCACTCAAAGAAAAGCCACCAGCTGTCTCT

GTGATCTGCTCATTCTGTGTAAACTTGTGTTCTCAACAATTAACAATTAACCAAAAGCCT

CTGGAGCTGCGTGAGCATCACGATGTGTTTGTCTTCTGTGAGCGTGGCAGTGTCCACGAA

GTCAATGTGAATTGGTTGGCCAAACACAGCTAATGTGGCATGTGTGATTGGACAGCCACT

TAAAAGTGTTAAAGTGTGTTTACGCACAGGATTGGTTTTCCGCTGAATCAGGCAAAACCA

GCAGAACCGATCTAAACTCGAGTATAAAAGTGAGTAGGAGATGCTATTCGTCCCAAAACA

TCTCCACCACCATATCCACCATTCCTCGCCTCTGCTTCGAGAACAGGTGAGTGTCGAGCG

GGATGGTAAATCTACAGAGAATGCGGAGTGTAGCTTGTGTACATGTTTTTGATTAACAGA

GATTGTATGTGTGTGTTTGTGTCAGTGTGA

```
ATGGACCTGCCTGACGACCACTACCTGTCCACCCAGACCATCCTGTCCAAGGACCTGAAC
 M  D  L  P  D  D  H  Y  L  S  T  Q  T  I  L  S  K  D  L  N

TCCGGACTCAGATCTGGCAGCGGTCTCGAGATGGAAGTTAGCGCTCTGGAAAAAGAAGTG
 S  G  L  R  S  G  S  G  L  E  M  E  V  S  A  L  E  K  E  V

TCTGCACTCGAGAAAGAAGTAAGTGCCCTTGAGAAGGAGGTGTCCGCACTCGAGAAGGAG
 S  A  L  E  K  E  V  S  A  L  E  K  E  V  S  A  L  E  K  E

GTCAGCGCCCTGGAAAAGGAAAAGCGAGACCATATGGTTTTGCTTGAGTATGTTACAGCG
 V  S  A  L  E  K  E  K  R  D  H  M  V  L  L  E  Y  V  T  A

GCTGGCATTACCGATGCATCAGGTGATGAAGTTGATGGTGGCGGTGGCAAGGTGTCCGCC
 A  G  I  T  D  A  S  G  D  E  V  D  G  G  G  G  K  V  S  A

CTGAAGGAAAAAGTAAGCGCACTGAAAGAAAAGGTGAGCGCGCTGAAGGAGAAAGTGAGC
 L  K  E  K  V  S  A  L  K  E  K  V  S  A  L  K  E  K  V  S

GCCCTGAAAGAGAAAGTCTCTGCCCTTAAGGAGGATATCGAGGGCAGAGGAAGTCTGCTA
 A  L  K  E  K  V  S  A  L  K  E  D  I  E  G  R  G  S  L  L

ACATGCGGTGACGTCGAGGAGAATCCTGGCCCAAAGCTTGCCACCATGCGCAAAGGCGAA
 T  C  G  D  V  E  E  N  P  G  P  K  L  A  T  M  R  K  G  E

GAACTGTTTACCGGCATTGTGCCGATTCTGGTGGAACTGGATGGCGATGTGAACGGCCAT
 E  L  F  T  G  I  V  P  I  L  V  E  L  D  G  D  V  N  G  H

AAATTTTTTGTGCGCGGCGAAGGCGAAGGCGATGCGACCATTGGCAAACTGAGCCTGAAA
 K  F  F  V  R  G  E  G  E  G  D  A  T  I  G  K  L  S  L  K

TTTATTTGCACCACCGGCAAACTGCCGGTGCCGTGGCCGACCCTGGTGACCACCCTGACC
 F  I  C  T  T  G  K  L  P  V  P  W  P  T  L  V  T  T  L  T

TATGGCGTGCAGTGCTTTAGCCGCTATCCGGATCATATGAAACGCCATGATTTTTTTAAA
 Y  G  V  Q  C  F  S  R  Y  P  D  H  M  K  R  H  D  F  F  K

AGCGCGATGCCGGAAGGCTATGTGCAGGAACGCACCATTTATTTTAAAGATGATGGCACC
 S  A  M  P  E  G  Y  V  Q  E  R  T  I  Y  F  K  D  D  G  T

TATAAAACCCGCGCGGAAGTGAAATTTGAAGGCGATACCCTGGTGAACCGCATTGAACTG
 Y  K  T  R  A  E  V  K  F  E  G  D  T  L  V  N  R  I  E  L

AAAGGCATTGATTTTAAAGAAGATGGCAACATTCTGGGCCATAAACTGGAATATAACTTT
 K  G  I  D  F  K  E  D  G  N  I  L  G  H  K  L  E  Y  N  F

AACAGCCATAAAGTGTATATTACCGCGGATAAACAGAACAACGGCATTAAAGCGAACTTT
 N  S  H  K  V  Y  I  T  A  D  K  Q  N  N  G  I  K  A  N  F

ACCATTCGCCATAACGTGGAAGATGGCAGCGTGCAGCTGGCGGATCATTATCAGCAGAAC
 T  I  R  H  N  V  E  D  G  S  V  Q  L  A  D  H  Y  Q  Q  N

ACCCCGATTGGCGATGGCCCGGTTCTTCTTCCTGGCGGCCGCTCTAGAGAGGGCAGAGGA
 T  P  I  G  D  G  P  V  L  L  P  G  G  R  S  R  E  G  R  G

AGTCTGCTAACATGCGGTGACGTCGAGGAGAATCCTGGCCCAGAATTGATGGTGAGCAAG
 S  L  L  T  C  G  D  V  E  E  N  P  G  P  E  L  M  V  S  K
```

```
GGCGAGGAGGATAACATGGCCATCATCAAGGAGTTCATGCGCTTCAAGGTGCACATGGAG
 G  E  E  D  N  M  A  I  I  K  E  F  M  R  F  K  V  H  M  E

GGCTCCGTGAACGGCCACGAGTTCGAGATCGAGGGCGAGGGCGAGGGCCGCCCCTACGAG
 G  S  V  N  G  H  E  F  E  I  E  G  E  G  E  G  R  P  Y  E

GGCACCCAGACCGCCAAGCTGAAGGTGACCAAGGGTGGCCCCCTGCCCTTCGCCTGGGAC
 G  T  Q  T  A  K  L  K  V  T  K  G  G  P  L  P  F  A  W  D

ATCCTGTCCCCTCAGTTCATGTACGGCTCCAAGGCCTACGTGAAGCACCCCGCCGACATC
 I  L  S  P  Q  F  M  Y  G  S  K  A  Y  V  K  H  P  A  D  I

CCCGACTACTTGAAGCTGTCCTTCCCCGAGGGCTTCAAGTGGGAGCGCGTGATGAACTTC
 P  D  Y  L  K  L  S  F  P  E  G  F  K  W  E  R  V  M  N  F

GAGGACGGCGGCGTGGTGACCGTGACCCAGGACTCCTCCCTGCAGGACGGCGAGTTCATC
 E  D  G  G  V  V  T  V  T  Q  D  S  S  L  Q  D  G  E  F  I

TACAAGGTGAAGCTGCGCGGCACCAACTTCCCCTCCGACGGCCCCGTAATGCAGAAGAAG
 Y  K  V  K  L  R  G  T  N  F  P  S  D  G  P  V  M  Q  K  K

ACCATGGGCTGGGAGGCCTCCTCCGAGCGGATGTACCCCGAGGACGGCGCCCTGAAGGGC
 T  M  G  W  E  A  S  S  E  R  M  Y  P  E  D  G  A  L  K  G

GAGATCAAGCAGAGGCTGAAGCTGAAGGACGGCGGCCACTACGACGCTGAGGTCAAGACC
 E  I  K  Q  R  L  K  L  K  D  G  G  H  Y  D  A  E  V  K  T

ACCTACAAGGCCAAGAAGCCCGTGCAGCTGCCCGGCGCCTACAACGTCAACATCAAGTTG
 T  Y  K  A  K  K  P  V  Q  L  P  G  A  Y  N  V  N  I  K  L

GACATCACCTCCCACAACGAGGACTACACCATCGTGGAACAGTACGAACGCGCCGAGGGC
 D  I  T  S  H  N  E  D  Y  T  I  V  E  Q  Y  E  R  A  E  G

CGCCACTCCACCGGCGGCATGGACGAGCTGTACAAGTAA
 R  H  S  T  G  G  M  D  E  L  Y  K  *
```

CTATTAATAGTGTGCATTCAGTGCAGGATGTTATGTATTTATTTTCTGTAGTGCAAACTT

TTTCAAAGAATTTTCCAGTCTAAGCTTTTCAGTCAAGAAAAAAAAAAAGCGATTGATTAT

CTGTGATATTTAAAAAAATTAGCTCTTAAATCTGGTGAACCAAGTGGCCACAACACATTA

AATACTGACGTTCTACAGCCATGTAGCTTCATCTGGTCTGGTTTGTTTTGGCAGGCACTT

ATTTTCCGGTGATAGTTGGCCACAAGAGTATTGTCTGAGCCATTCAGTGCTAGACTGTCA

TTCTCAGGTCAGAGTCCACCCCGCTGATTCTGCTGACAACACTGTTCCCACCATGAGATA

ATGCCATTCCAGAGAGATCCATTTGTAAGCCCCTCTTTCTGCAGCACAGGTATATAACCA

GGGGTCTGCCTCCACTAAGGCCGGCACACATCATTTGGGGATCTTTGTACTGTACTAGCC

TCTATTAGCCTCCTACTTGCGTTCAGTTCACATCTTTTGTTCATCAGGTGCAATAAGTTA

CAGTAAGGAGTTACCAGGTCTGACAACATGGTGAGCAAGGGCGAGGAGCTGTTCACCGGG
M V S K G E E L F T G

GTGGTGCCCATCCTGGTCGAGCTGGACGGCGACGTAAACGGCCACAAGTTCAGCGTGTCC
V V P I L V E L D G D V N G H K F S V S

GGCGAGGGCGAGGGCGATGCCACCTACGGCAAGCTGACCCTGAAGCTGATCTGCACCACC
G E G E G D A T Y G K L T L K L I C T T

GGCAAGCTGCCCGTGCCCTGGCCCACCCTCGTGACCACCCTGGGCTACGGCCTGCAGTGC
G K L P V P W P T L V T T L G Y G L Q C

TTCGCCCGCTACCCCGACCACATGAAGCAGCACGACTTCTTCAAGTCCGCCATGCCCGAA
F A R Y P D H M K Q H D F F K S A M P E

GGCTACGTCCAGGAGCGCACCATCTTCTTCAAGGACGACGGCAACTACAAGACCCGCGCC
G Y V Q E R T I F F K D D G N Y K T R A

GAGGTGAAGTTCGAGGGCGACACCCTGGTGAACCGCATCGAGCTGAAGGGCATCGACTTC
E V K F E G D T L V N R I E L K G I D F

```
AAGGAGGACGGCAACATCCTGGGGCACAAGCTGGAGTACAACTACAACAGCCACAACGTC
 K  E  D  G  N  I  L  G  H  K  L  E  Y  N  Y  N  S  H  N  V

TATATCACCGCCGACAAGCAGAAGAACGGCATCAAGGCCAACTTCAAGATCCGCCACAAC
 Y  I  T  A  D  K  Q  K  N  G  I  K  A  N  F  K  I  R  H  N

ATCGAGGACGGCGGCGTGCAGCTCGCCGACCACTACCAGCAGAACACCCCCATCGGCGAC
 I  E  D  G  G  V  Q  L  A  D  H  Y  Q  Q  N  T  P  I  G  D

GGCCCCGTGCTGCTGCCCGACAACCACTACCTGAGCTACCAGTCCGCCCTGAGCAAAGAC
 G  P  V  L  L  P  D  N  H  Y  L  S  Y  Q  S  A  L  S  K  D

CCCAACGAGAAGCGCGATCACATGGTCCTGCTGGAGTTCGTGACCGCCGCCGGGATCACT
 P  N  E  K  R  D  H  M  V  L  L  E  F  V  T  A  A  G  I  T

CTCGGCATGGACGAGCTGTACAAGTAA
 L  G  M  D  E  L  Y  K  *
```

ESM Tables

ESM Table 1: Transgenic zebrafish lines:

**Brief line name**	**Full zebrafish line name**
Tg(elaA:NTR;ins:GFP)	Tg (elaA:myrpalmDEVD-mScarlet-NTR;cryaA:Venus)
Tg(elaA:H2B-mScarlet)	Tg(elaA:H2B-mScarlet;cryaA:Venus)
Tg(ins:XBP1v)	Tg(ins:H2B-mTurquoise2-xbp1-Venus;cryaA;mCherry)
Tg(ins:FlipGFP)	Tg(ins:FlipGFP-mCherry;cryaA:Venus)

ESM Table 2: Double-crossed transgenic zebrafish lines:

**Double Tg line**	**Line I**	**Line II**
Tg(elaA:NTR;ins:GFP)	Tg(elaA:myrpalmDEVD-mScarlet-NTR;cryaA:Venus)	Tg(ins:GFP)
Tg(elaA:NTR;ins:XBP1v)	Tg(elaA:myrpalmDEVD-mScarlet-NTR;cryaA:Venus)	Tg(ins:H2B-mTurquoise2-xbp1-Venus;cryaA;mCherry)
Tg(elaA:H2B-mScarlet;ins:XBP1v)	Tg(elaA: H2B-mScarlet;cryaA:Venus)	Tg(ins:H2B-mTurquoise2-xbp1-Venus;cryaA;mCherry)
Tg(elaA:NTR;ins:FlipGFP)	Tg(elaA:myrpalmDEVD-mScarlet-NTR;cryaA:Venus)	Tg(ins:FlipGFP-mCherry)

ESM Table 3: Addgene plasmids that were used to generate the transgenic lines:

**Addgene number**	**More information**	**References**
# 84032	pHD066 was a gift from Daniel Hesselson & Didier Stainier (http://n2t.net/addgene:84032)	[1]
# 14867	MyrPalm-CFP was a gift from Alexandra Newton (http://n2t.net/addgene:14867)	[2]
# 66593	ins:CFP-NTR was a gift from Didier Stainier (http://n2t.net/addgene:66593)	[3]
# 124431	PCS2-FlipGFP (Casp3 cleavage seq) T2A mCherry was a gift from Xiaokun Shu (http://n2t.net/addgene:124431)	[4]

ESM Table 4: Primers that were used in plasmid cloning:

Primer	Sequence
BglII-*cryaA*	5'-GGTGGT AGATCT ATTAATAGTGTGCATTCAGTGCAG-'
NcoI-bGH	5'-AATAAT CCATGG GCGATCTCTCCCCAGCATG-3'
ApaI-ElaA	5'-GTATTAGGGCCC AAGCTTTTTCCAGTGTACTTACC-3'
NheI-ElaA	5'-CTAATTGCTAGC CAACACTGGCATCAACTGC-3'
KpnI-MyrPalm	5'-ATAATAGGTACCGCCACCATGGGCTGCATC-3'
BamHI-myrpalm(G4748E)	5'-AACTTTGGATCCAAGTCCACCTCGTCGTCG-3'
pcDNA3.1	5'-TAATACGACTCACTATAGGGA-3'
ClaI STOPmScarlet(-NotI)	5'-GCGCATCGATTTACTTGTACAGCTCGTCC-3'
NotI-NTR	5'-TGTTATGCGGCCGCTATGGACATCATCTCTGTGG-3'
XbaI-NTR	5'-CACCGCTCTAGATTACACCTCTGTCAGGGT-3'
ApaI-ins	5'-GTATTAGGGCCCTTCAGCATTGAACTCTATTGTG -3'
NheI-ins	5'-CTAATTGCTAGC GTCACACTGACACAAACACAC-3'
Nhe1 Flip-GFP	5'-TAAGACGCTAGCATCCGCCACCATGGACCTG-3'
Cla1 Flip-GFP	5'-TGCGCCATCGATCTTGAATTCTTACTTGTACAG-3'

ESM Table 5: QPCR primers:

qPCR primers	Primer sequence
ela3l-FW	GTTGTCGCTGGATGCAATGGAG
ela3l-REV	TGCCGTCAGAGTTCTTGCAGTTC
trypsin-FW	ACCAGCTGTCTGATCTCTGGATGG
trypsin-REV	CAGACGGCTTGGGTAATTGCTTC
18s-fw	GCGTTGATTAAGTCCCTGCC
18s-rev	GTTTGATCGTCTTCTCGGCG

References

1 Hesselson D, Anderson RM, Stainier DYR (2011) Suppression of Ptf1a activity induces acinar-to-endocrine conversion. Current Biology 21:712–717. https://doi.org/10.1016/j.cub.2011.03.041

2. Violin JD, Zhang J, Tsien RY, Newton AC (2003) A genetically encoded fluorescent reporter reveals oscillatory phosphorylation by protein kinase C. Journal of Cell Biology 161:899–909. https://doi.org/10.1083/jcb.200302125

3. Curado S, Anderson RM, Jungblut B, et al (2007) Conditional targeted cell ablation in zebrafish: A new tool for regeneration studies. Developmental Dynamics 236:1025–1035. https://doi.org/10.1002/dvdy.21100

4. Zhang Q, Schepis A, Huang H, et al (2019) Designing a Green Fluorogenic Protease Reporter by Flipping a Beta Strand of GFP for Imaging Apoptosis in Animals. J Am Chem Soc 141:4526–4530. https://doi.org/10.1021/jacs.8b13042

ESM Figures

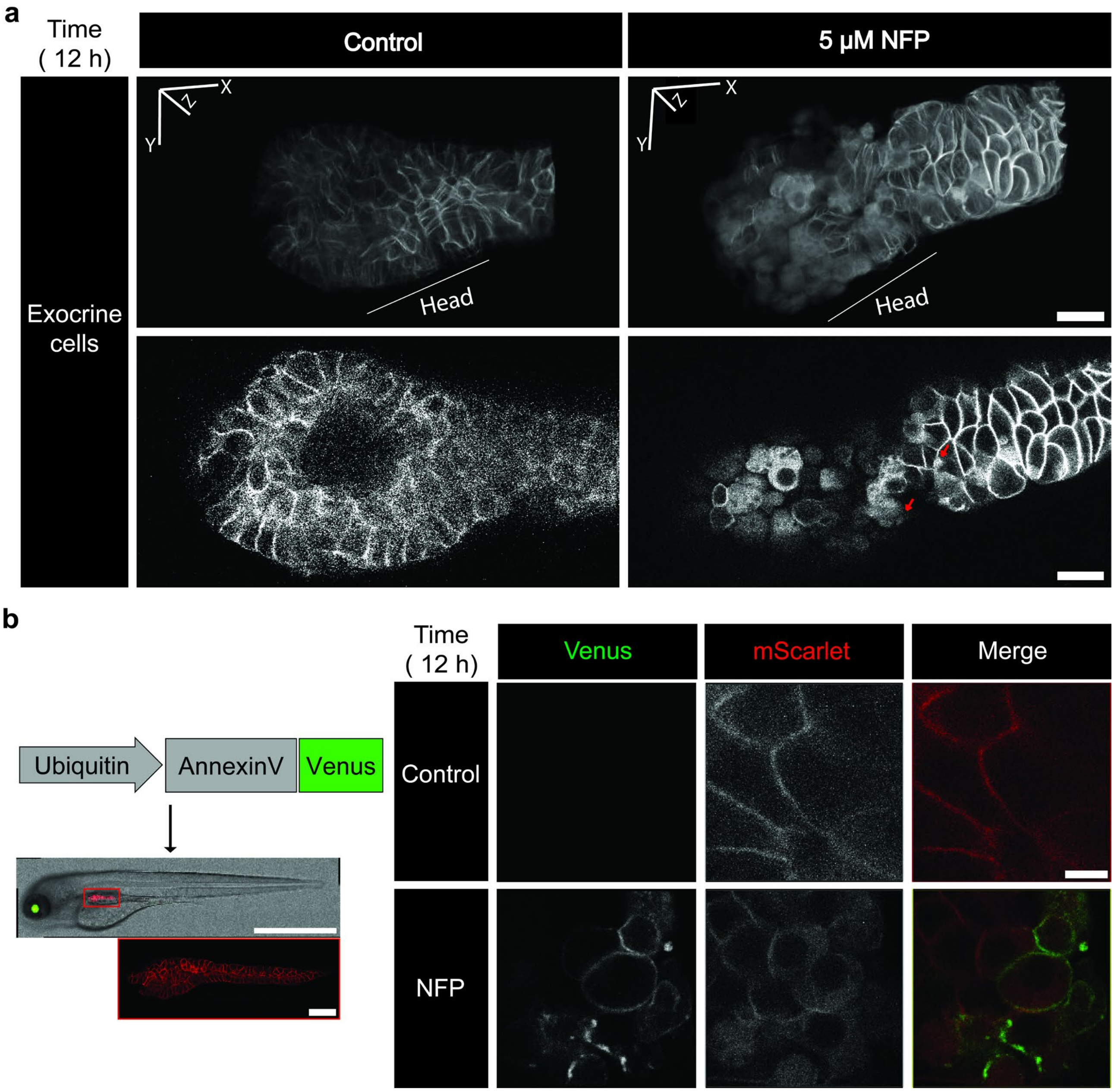

ESM Fig. 1: Heterogeneity of exocrine cell damage.

a: The first row displays representative confocal data of mScarlet signal, showing the heterogeneity of mScarlet translocation within a single zebrafish pancreas, especially in the pancreas head, compared to control. The second row shows confocal images with red arrows referring to some apoptotic characteristics like membrane blebbing. Bar: 50 μm. **b**: The apoptotic reporter, Annexin V fused with Venus under a general promoter (Ubiquitin), is transiently expressed in Tg(elaA:NTR) at 120 hpf. Bars: 1 mm, and 50 μm. Confocal data shows apoptotic cytosolic exocrine cells and annexin V- Venus+ plasma membrane after 12 hours of 5 μM NFP treatment. Bar: 5 μm.

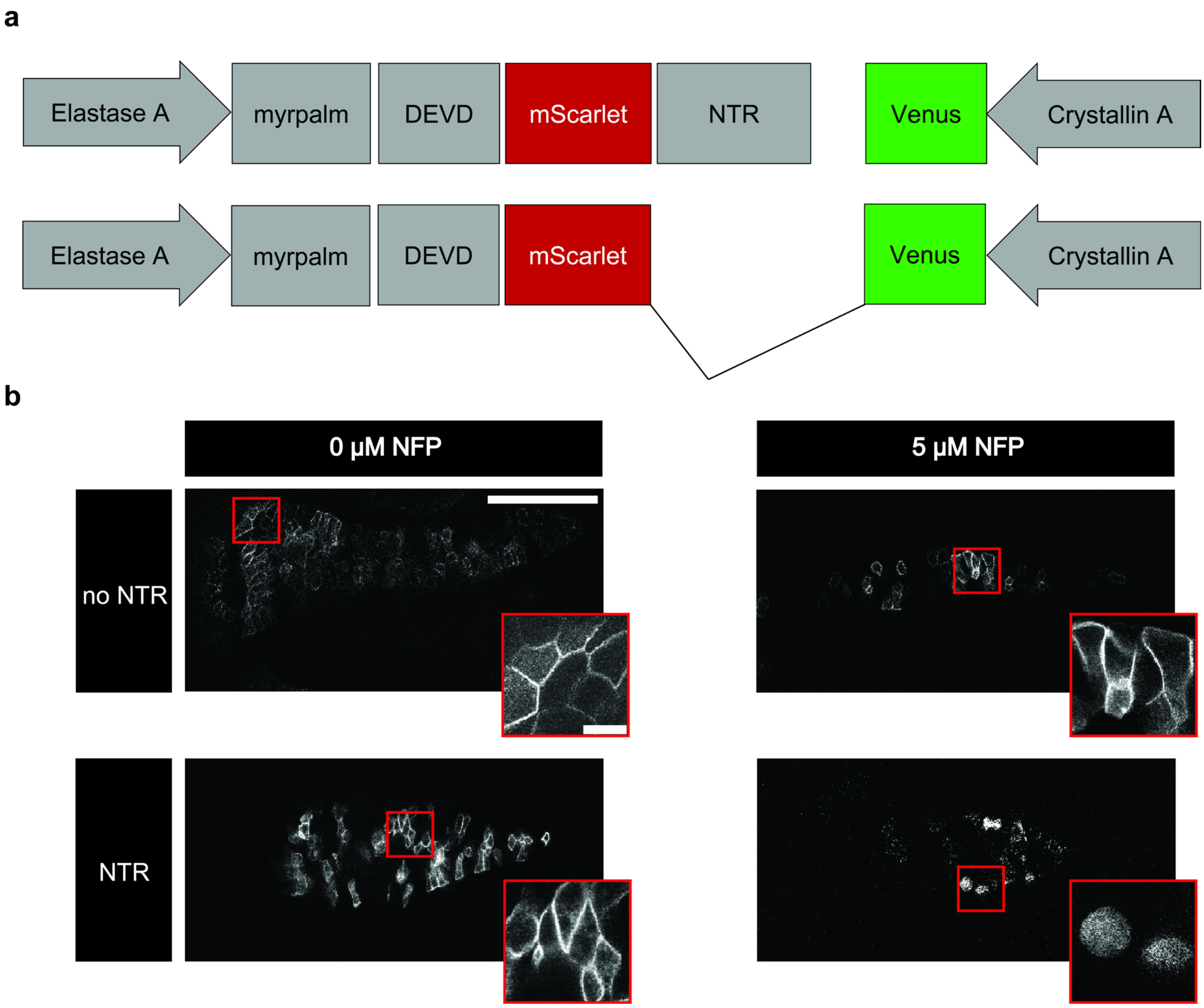

ESM Fig. 2: NFP toxicity is NTR dependent.

a: Testing two similar constructs with or without NTR. **b**: Confocal images, showing mScarlet signal after 12 hours administration of 5 µM NFP or no NFP on NTR positive or negative exocrine cells in zebrafish larvae at 120 hpf. Bars: 500, and 50 µm.

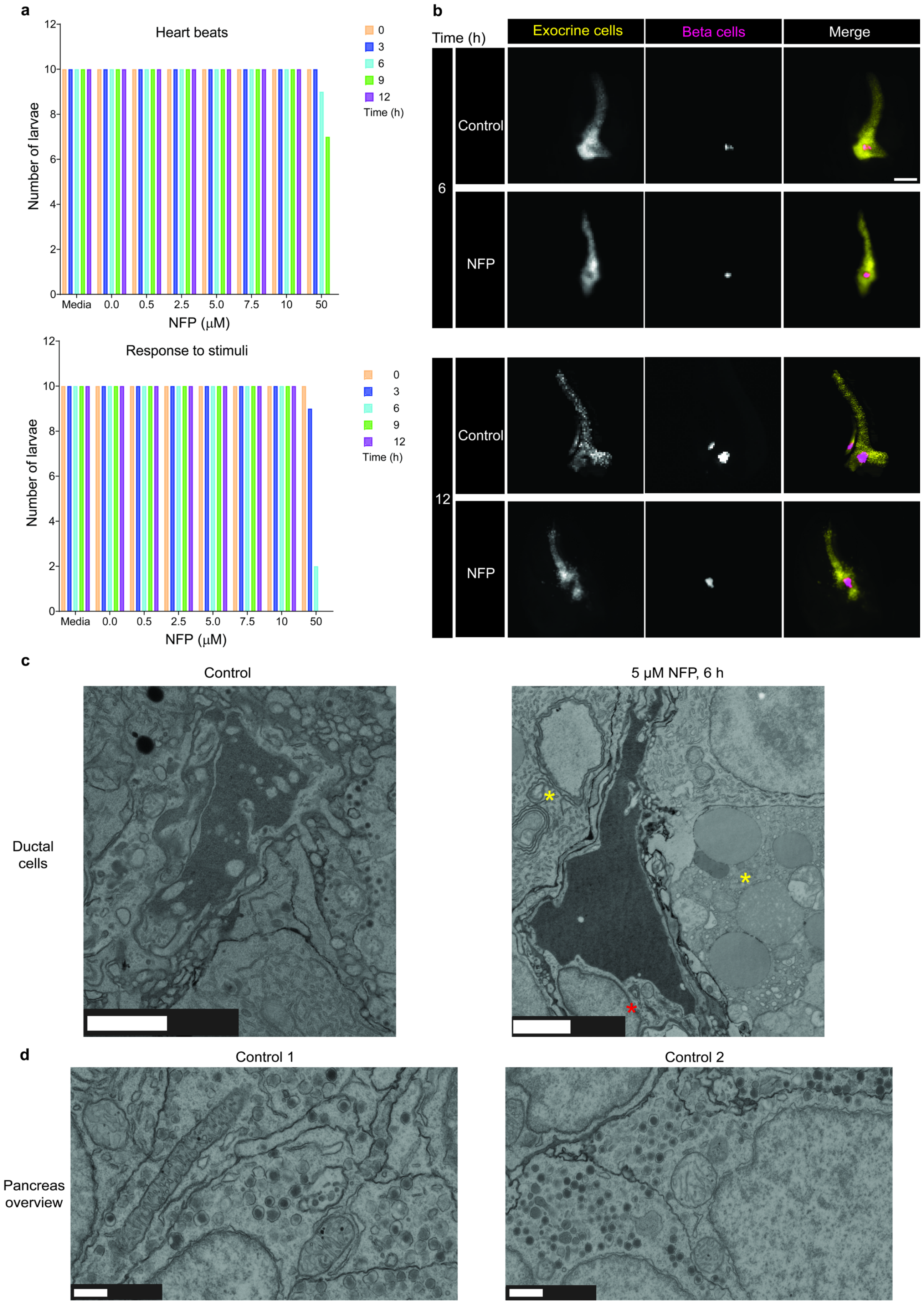
a
Heart beats
Number of larvae
NFP (μM)
Media
0.0
0.5
2.5
5.0
7.5
10
50
Time (h)
Response to stimuli
b
Time (h)
Exocrine cells
Beta cells
Merge
Control
NFP
6
12
c
Control
5 μM NFP, 6 h
Ductal cells
d
Control 1
Control 2
Pancreas overview

ESM Fig. 3: NFP toxicity and pancreas volume in transgenic zebrafish larvae.

a: The toxicity of NFP (measured by heart rate and response to stimuli) on wild-type AB zebrafish larvae after NFP treatment at concentrations of 0.5, 2.5, 5, 7.5, 10, and 50 μM for durations of 0, 3, 6, 9, and 12 hours, with a media-only control and 0 μM NFP in 0.1% DMSO control (n=10 per group). **b**: Representative images captured using light sheet fluorescence microscopy and processed to measure pancreas volume. Bar: 50 μm. **c**: Representative EM images of ductal cells from 6 hours post-NFP treatment and control (0 μM NFP in 0.1% DMSO). Yellow asterisks refer to abnormal exocrine cells and red asterisk refers to ductal cells in the NFP treated condition. Bar: 2 μm. **d**: EM overview of pancreas region in two controls; Control 1 refers to 5 μM NFP treated Tg(ins:GFP) larvae and Control 2 refers to 5 μM NFP treated AB larvae. Bar: 500 nm.

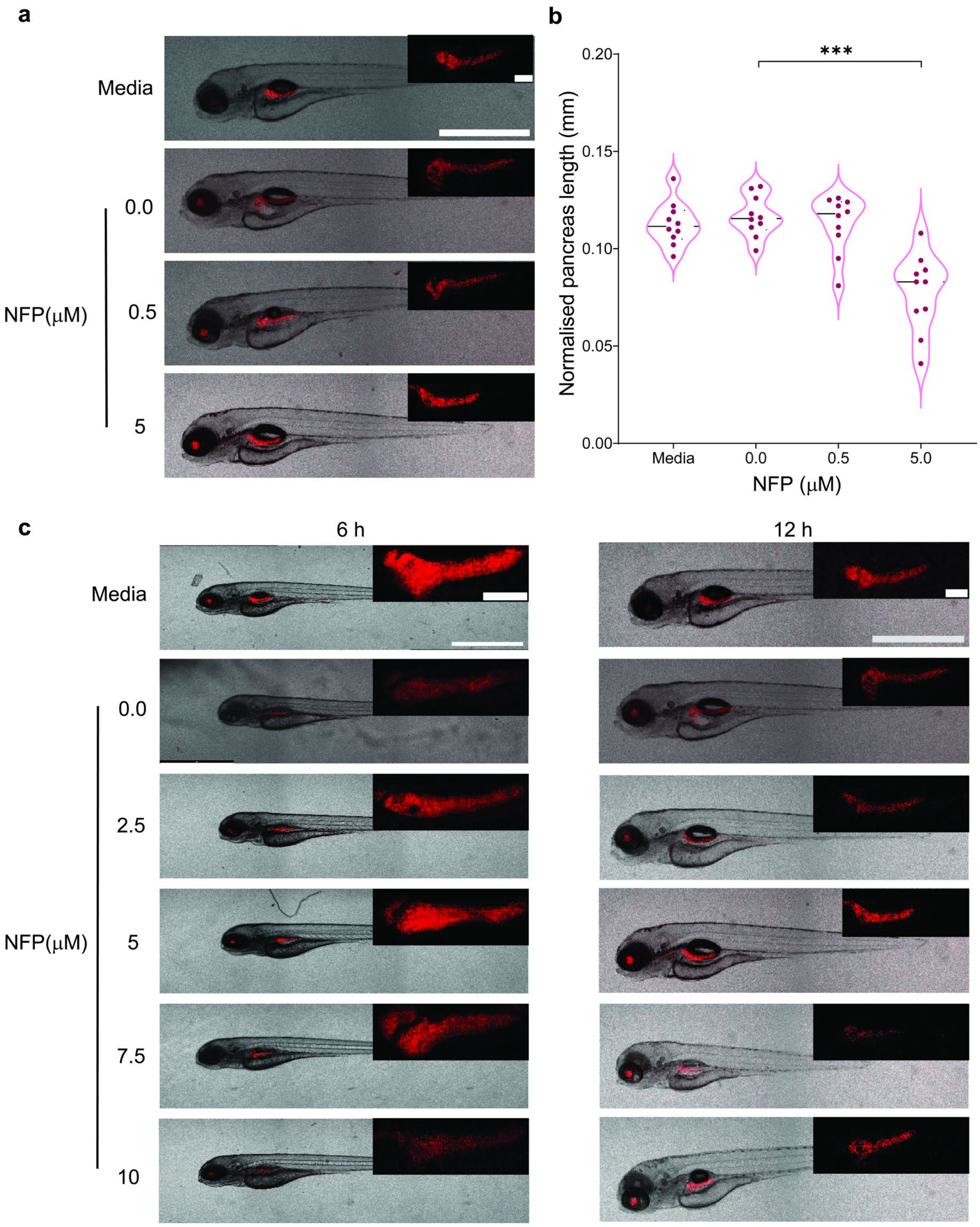

ESM Fig. 4: NFP optimization in transgenic zebrafish larvae.

a-b: Evaluation of NFP concentrations for a 12-hour treatment period on pancreas length after normalization with the entire length of transgenic larvae. Bars: 1 mm, 100 µm. Unpaired t test was used for statistical differences between groups. *P-value <0.05, **P-value <0.01, ***P-value <0.001. **c**: Dynamic treatment effect of various concentrations of NFP on pancreas length in transgenic larvae (n=6 to 8 per group). Bars: 1 mm, and 100 µm. 0 µM NFP in 0.1% DMSO as a control. Images shown in ESM Fig. 4a are the same in ESM Fig. 4c.

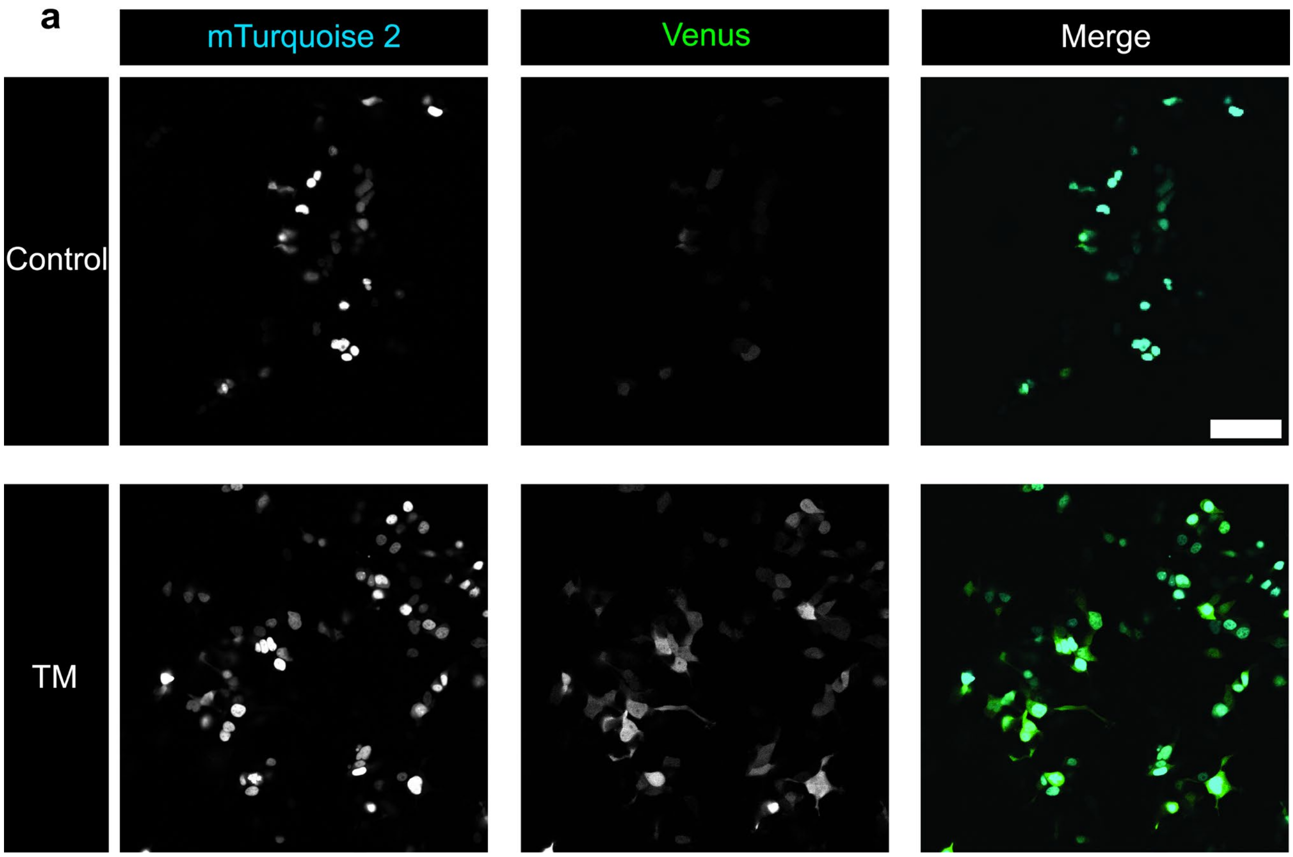

ESM Fig. 5: Validation of ER stress reporter *in vitro*.

a: Confocal images, showing transient expression of the xbp1Venus ER stress reporter in HEK293T cells after 6 hours treatment with 2 µg/ml tunicamycin (TM). Bar: 200 µm.

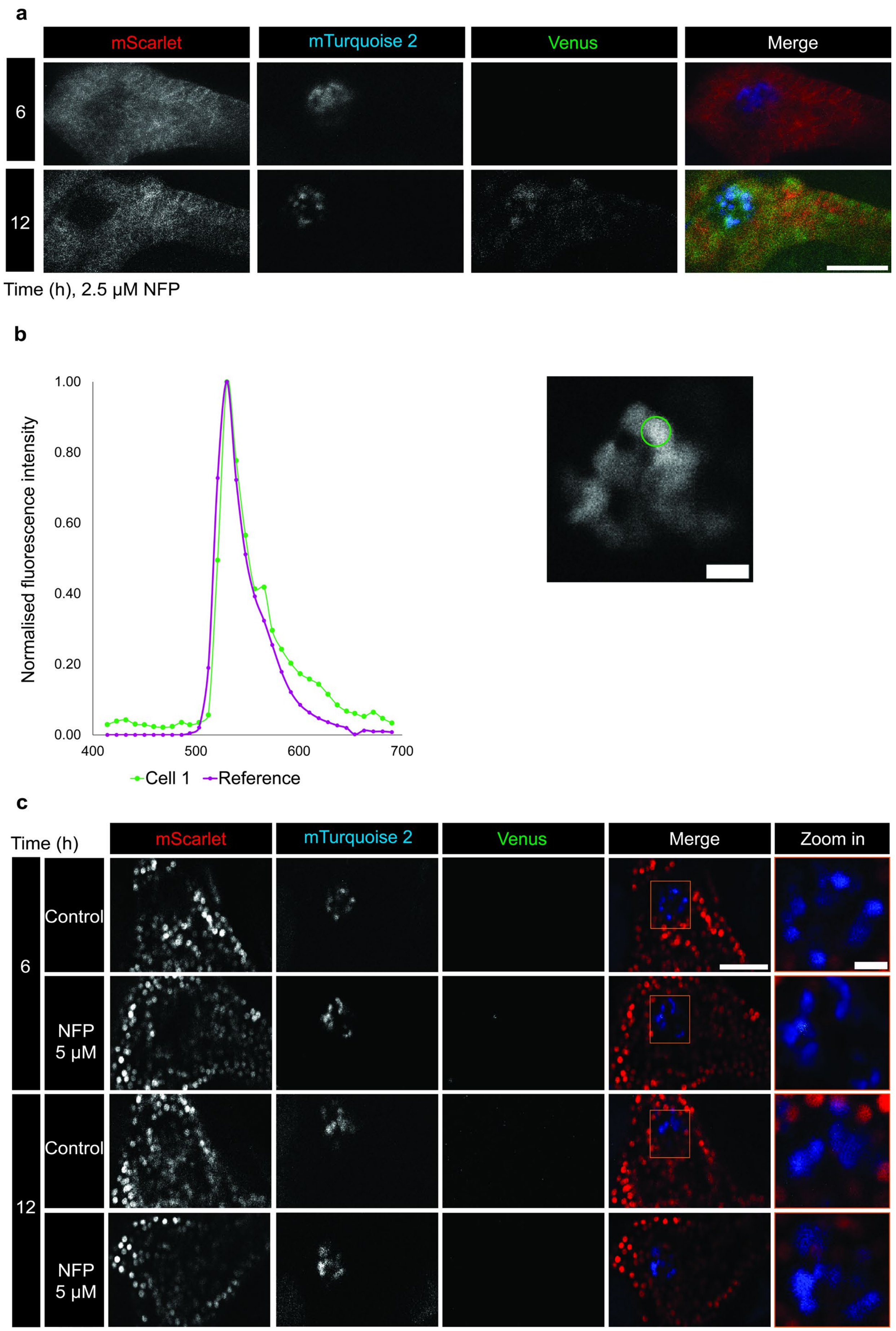
a
mScarlet
mTurquoise 2
Venus
Merge
6
12
Time (h), 2.5 μM NFP
b
1.00
0.80
0.60
0.40
0.20
0.00
Normalised fluorescence intensity
400
500
600
700
Cell 1
Reference
c
Time (h)
mScarlet
mTurquoise 2
Venus
Merge
Zoom in
6
Control
NFP 5 μM
12
Control
NFP 5 μM

ESM Fig. 6: Assessment of ER stress reporter in zebrafish larvae.

a: The expression of mTurquoise2 and Venus in beta cells, and mScarlet in exocrine cells, following treatment with 2.5 μM NFP for 6 and 12 hours. Bar: 50 μm. **b**: Lambda scan shows the presence of Venus in beta cells after treating zebrafish with NFP; Cell 1 line compared to the reference line. Bar: 10 μm. Venus emission spectra is found on https://www.fpbase.org/protein/venus/. **c**: Evaluating NFP effects on mturquoise2+ beta cells and NTR- exocrine cells in outcross Tg(elaA:H2B-mScarlet) with ER stress reporter line Tg(ins:XBP1v). Bars: 50, and 10 μm.

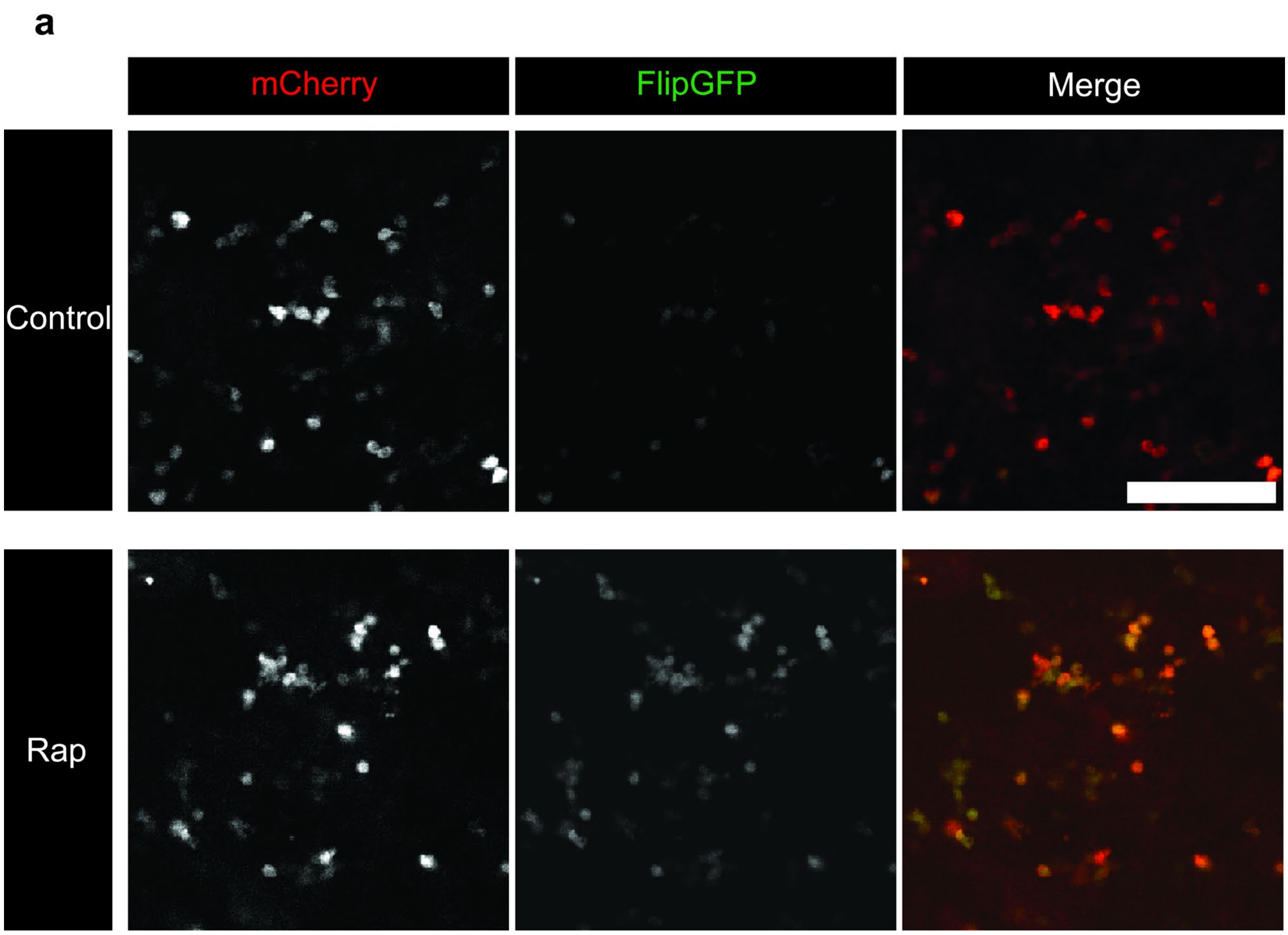

ESM Fig. 7: In vitro validation of Flip-GFP reporter.

a: Confocal images showing mCherry and Flip-GFP signals after transient transfection of pcDNA-FlipGFP-mCherry in control and Raptinal (Rap) treated HEK293T cells (1 μM for 6 hours).

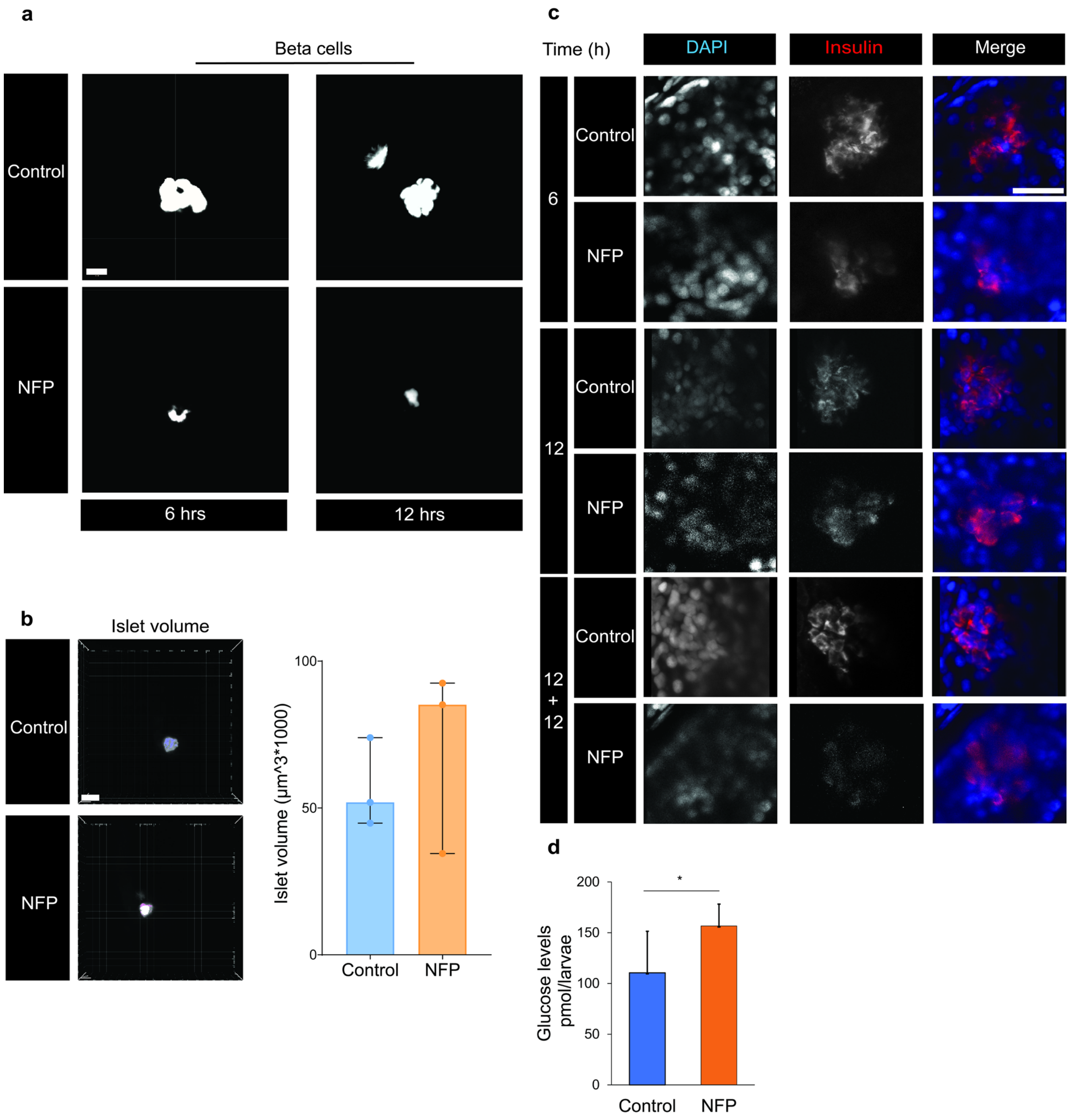

ESM Fig. 8: Islet volume and insulin immunostaining post-NFP treatment.

a: Representative images of GFP signal captured using light sheet fluorescence microscopy and processed to measure islet volume in Tg(elaA:NTR;ins:GFP) after 5 µM NFP treatment. Bar: 20 µm. **b**: Representative light sheet data of GFP signal and bar graph, showing the islet volume after 12 hours of NFP treatment in Tg(ins:GFP) larvae at 5 dpf (n=3). Bar: 50 µm. Data represent the median values with bars indicating the range from the maximum to the minimum data points. Unpaired t test was used for statistical differences between groups. **c**: Maximum projection images of insulin immunostaining with DAPI staining in whole larvae following NFP treatment and controls (n=5). Bar: 25 µm. **d**: Glucose concentration levels in 12 hours of 5 µM NFP treated Tg(elaA:NTR;ins:GFP) vs. control (0 µM NFP in 0.1% DMSO) at 96 hpf. Error bar=SD. One-sided t test was used for statistical differences between groups. *P-value <0.05, **P-value <0.01, ***P-value <0.001.

ESM Videos

ESM Video 1 : 3D volume of zebrafish pancreas at 107 hpf. Yellow refers to pancreas and magenta refers to islet.

ESM Video 2: Dynamic exocrine cell response after damage induction overtime. Interval: 1 hour for 12 hours.

ESM Video 3: *In vivo* monitoring of apoptosis in mCherry+ beta cells overtime in NFP-Treated condition.

Intervals: 1 hour, duration: 13 hours, beta nuclei and exocrine membranes are displayed in gray and apoptotic cells (if any) in green.

ESM Videos are accessible via http://www.nanotomy.org
